# Are gender-science stereotypes barriers for women in science, technology, engineering, and mathematics? Exploring when, how, and to whom in an experimentally-controlled setting

**DOI:** 10.3389/fpsyg.2023.1219012

**Published:** 2023-08-09

**Authors:** Alba Sebastián-Tirado, Sonia Félix-Esbrí, Cristina Forn, Carla Sanchis-Segura

**Affiliations:** Departament de Psicologia Bàsica, Clínica i Psicobiologia, Universitat Jaume I, Castelló de la Plana, Spain

**Keywords:** gender stereotypes, stereotype threat, math, implicit association test, STEM persistence

## Abstract

Based on Social Cognitive Career Theory principles, the present study sought to investigate whether stereotype threat experiences could act as a barrier and reduce the persistence of women in math-intensive activities. More specifically, we assessed whether the experimental activation of stereotypes about women’s lower math capabilities affected the performance, persistence, and self-selected difficulty of engineering students in a math task which required sustained effort. We also evaluated the relationships between these effects and the participants’ pre-testing gender-science stereotypes and math self-concept. A sample of 340 engineering students (175 and 165 self-identified as males and females, respectively) were recruited and randomly assigned to a control (Con) or stereotype threat (StA) condition to form four similarly sized groups. All participants rated their self-concept in four academic domains (math, chemistry, physics, and coding), completed the gender-science Implicit Association Test, and a “reading comprehension task” that served to promote the stereotype threat manipulation immediately before facing a modified version of the Math Effort Task (M-MET). We observed that, in the control condition, M-MET performance, self-selected difficulty, and persistence were similar in female and male participants, independent of their gender-science implicit stereotypes but correlated to their math self-concept scores. In contrast, the StA condition triggered opposite effects in female and male students, so they showed decreased/enhanced M-MET performance and self-selected difficulty, respectively. This experimental condition also resulted in enhanced persistence of the male students without affecting the number of trials completed by female students. These effects were correlated with the strength of the participants’ gender-science implicit stereotypes but not with their math self-concept scores. In fact, as revealed by finer-grain analyses, stereotype threat only had a significant impact on individuals harboring stereotypical gender-science implicit associations. Therefore, it is concluded that: (1) stereotypes can promote differences between male and female engineering students in their performance, self-confidence, and persistence in math-related activities; (2) These effects seem to be more prominent in individuals implicitly perceiving science as a masculine domain. The relevance of these findings to explain women’s enhanced abandonment rates of math-intensive studies is discussed.

## 1. Introduction

Despite the notable advances toward social equity between women and men in the last decades, there is a major gender-related asymmetry in certain areas of academic and professional specialization that results in an underrepresentation of women in Science, Technology, Engineering, and Mathematics (STEM) studies and professions. Thus, although in elementary, middle, and high school, girls and boys take math and science courses in roughly equal numbers, only around 20–25% of STEM students are women, a number that declines even further in subsequent educational stages and in the workplace (Hill et al., 2010; Manske, 2010; European Commission, 2016; OECD, 2017).

The underrepresentation of women in STEM studies severely increases their risk of exclusion and/or inequality in the labor market given that STEM related careers are expected to grow several times faster than the average rate of all occupations (National Science Board, 2010) and are among the best paid jobs (National Association of Colleges and Employers, 2009; European Commission, 2016). Not only is this a problem for women, but it is a waste of talent for the STEM field (Blickenstaff, 2006; European Commission, 2014; Noland et al., 2016) and also an economic cost for society as a whole. In fact, the supply of STEM talent is not expected to meet the market demand (Hewlett et al., 2008; Fayer et al., 2017), and it has been estimated that closing the gender gap in the STEM field would increase the EU GDP *per capita* by 0.7–0.9% in 2030 and by 2.2–3.0% in 2050 (Morais Maceira, 2017). Accordingly, the gender segregation that characterizes the STEM field at the educational and professional level is seen with increasing social and institutional concern (Hill et al., 2010; European Commission, 2016).

### 1.1. Explaining the underrepresentation of women in STEM studies/careers: a social cognitive career theory perspective

Social Cognitive Career Theory (SCCT; Lent et al., 1994, 2000) has probably been one of the most widely used theoretical frameworks when attempting to explain the reduced entrance, enhanced attrition, and high drop-out rate of women in STEM studies (Rottinghaus et al., 2018). Following Bandura’s general social cognitive theory (Bandura, 1977, 2000), on which it is based, SCCT hypothesizes that ability beliefs and confidence to perform well in specific cognitive/academic domains, as well as the anticipation of positive consequences derived from engaging in these activities, play a central role in the development of career and academic interests.

Ability beliefs/self-perceptions are built upon past self- and vicarious experiences, emotional reactions, and social persuasion/other people’s opinions (Bandura, 1977; Bandura et al., 1999), and they are formally referred to as *self-efficacy*. Self-efficacy is not a unitary or general construct akin to self-esteem or other similar traits, but a series of domain-specific sets of perceptions and beliefs that operate -and can be measured- at different levels of specificity (Pajares, 1996; Lent and Brown, 2006; Parsons et al., 2009). In this regard, an individual can express high self-efficacy in a cognitive/academic domain but low in others, and within that single domain, the same individual can feel confident and competent to successfully perform some tasks but not others. Self-efficacy beliefs/perceptions are not static either, and their dynamism is directly related to their level of specificity. Thus, general self-efficacy measures quite stable mental statements or representations about the self-perceived competence in a whole cognitive/academic domain (e.g., math) that are often referred to as the “*overall confidence*” (Parsons et al., 2009) or the “*self-concept*” in that domain (Pajares and Miller, 1994; Pajares, 1996). Conversely, measures of self-efficacy that are task- or even item-specific are much more malleable and can be dynamically moderated by environmental cues, so individuals may express high self-efficacy when facing a particular task in some contexts or under some circumstances but less so in others (Pajares and Miller, 1994; Pajares, 1996).

Therefore, self-efficacy -or, to better say, some of its percepts and measures- integrate the effects of past experience but also those of concurrent contextual factors. Depending on whether they facilitate or impede career-related decisions and behaviors, SCCT classifies influential contextual cues as *supports* (also referred to as *affordances*) or as *barriers*, respectively (Lent et al., 1996, 2000). Supports and barriers can be documented and also perceived as aspects of the environment but, regardless of whether or not they are grounded in reality, both have a major impact on self-efficacy, performance, and goals selection (Lent et al., 1994, 1996; Albert and Luzzo, 1999). More specifically, SCCT proposes that supports and barriers directly predict self-efficacy and that, mainly through this construct, they also predict the choice of goals and actions (Lent et al., 2002, 2003, 2015; Brown et al., 2008).

According to SCCT, gender differences in math self-efficacy could provide a reasonable explanation to the reduced number of women entering engineering and other STEM studies. In this regard, research has shown that (1) several measures of math self-efficacy predict math performance and the development of initial STEM-related interests and career goals (e.g., Hackett and Betz, 1989; Multon et al., 1991; Brown et al., 2008; Schunk and Pajares, 2009; Byars-Winston et al., 2010; Skaalvik et al., 2015); (2) despite having similar math abilities and grades (Lindberg et al., 2010; Else-Quest et al., 2013; Voyer and Voyer, 2014), boys and men tend to express higher self-efficacy than girls and women (Schunk and Pajares, 2002; Else-Quest et al., 2013; Zander et al., 2020; Mejía-Rodríguez et al., 2021); (3) women choosing STEM majors report higher levels of math self-efficacy than those opting for other studies (Hackett, 1985; Buschor et al., 2014) and, at least in some cases, similar to those exhibited by male STEM students (e.g., Brainard and Carlin, 1998; Macphee et al., 2013; Whitcomb et al., 2020). Taken together, these findings suggest that high levels of math self-efficacy are a pre-requirement to enter STEM studies and also that, despite having similar math abilities, less girls than boys complete these studies during their pre-major education years and, therefore, are less likely to choose STEM careers.

Similarly, SCCT proposes that, by determining interests and satisfaction, experience-dependent changes in self-efficacy affect academic persistence (Lent et al., 2013, 2015). Thus, different academic experiences and their effects on self-efficacy could explain why women leave STEM studies/professions at higher rates than men (Chen, 2013; Isphording and Qendrai, 2019; Clark et al., 2021; González-Pérez et al., 2022). In this regard, several studies have shown that self-efficacy is strongly related to academic persistence (Robbins et al., 2004; Wright et al., 2013) and also that math and science self-efficacy specifically predict persistence in STEM studies (e.g., Brown et al., 2008; Ackerman et al., 2013; Perez et al., 2014; Lee et al., 2015; Lent et al., 2015).

However, self-efficacy, by itself, does not suffice to adequately explain initial academic choices and persistence in their pursuit. As pointed out by Brown and Lent (1996), even those individuals with high levels of career self-efficacy, and career-aligned interests and goals may still avoid entering or pursuing a particular career if they perceive unbeatable barriers to attaining their academic/professional goals. Therefore, barriers, supports, and self-efficacy are supposed to play similarly important roles in career-related choices (Lent et al., 1994, 2000; Brown et al., 2008) and in the SCCT-based attempts to explain the underrepresentation of women in STEM studies/professions. However, research in this area has been less conclusive. Although some barriers and supports have been identified, most of these contextual factors seem to be domain-unspecific and/or do not seem to differentially affect math self-efficacy in male and female students (Lent et al., 2000; Fouad et al., 2002, 2010; Lent and Brown, 2006; Byars-Winston and Fouad, 2008; Miller M. J. et al., 2015). Accordingly, calls for identifying other possible barriers and supports and for improving their measurement have been made, as those traditionally assessed are “*typically too broad to offer much precision in predicting domain-specific criteria*” (Lent and Brown, 2006, p. 30) and the self-report instruments employed to evaluate them are largely dependent on the students’ awareness, ability or willingness to disclose them (Fouad et al., 2010).

### 1.2. Stereotypes as barriers for women in STEM studies

Gender-related stereotypes have been long proposed as a major factor underlying the underrepresentation of women in STEM studies and professions and, from a SCCT perspective, they can be conceptualized as distal and proximal barriers to their careers in this field. Distal barriers are environmental influences (or their perceptions) that are instrumental in determining the learning experiences through which overall self-efficacy, interests, and outcome expectations for particular academic domains are progressively forged (Lent et al., 1994). On the other hand, proximal barriers are contemporary contextual influences (or their percepts) that, by affecting more specific forms of self-efficacy, can moderate the relationships between already established interests and goals as well as between goals and behaviors, especially under adverse conditions (Lent et al., 2001).

Stereotypes about women’s lower math abilities and about the STEM field itself lead to the socially shared perception of math and “hard sciences” as “male” domains (Nosek et al., 2010; Nosek and Smyth, 2011; Cheryan et al., 2015; Makarova and Herzog, 2015). These stereotypical views are already held by six-year-old children (Cvencek et al., 2011; Miller D. I. et al., 2015; Master et al., 2021) but also by their parents and teachers, who also bear different expectations, attributions, and evaluations of girls’ and boys’ math abilities and achievements, and tend to grant them different opportunities and support in their math-related experiences (Eccles et al., 1990; Beilock et al., 2010; Gunderson et al., 2012). This results in a reduction of girls’ math self-concept (Eccles et al., 1990; Beilock et al., 2010; Eccles, 2011; Nosek and Smyth, 2011; Steffens and Jelenec, 2011; Garriott et al., 2017; Hand et al., 2017) and, thereby, to a reduced interest, motivation, and performance in math that ultimately preclude their chances of joining STEM studies (Lent et al., 1994; Nagy et al., 2007; Nosek and Smyth, 2011; Garriott et al., 2017; Wang and Degol, 2017; Luo et al., 2021). Conversely, being favored by stereotypes and a higher math self-concept, boys become more prone to join engineering and other math-intensive studies. Accordingly, empirical evidence shows that women entering STEM majors usually have a higher math self-concept (Hackett, 1985; Buschor et al., 2014) and weaker gender-science stereotypes (Nosek and Smyth, 2011; Smeding, 2012; Smyth and Nosek, 2015; Sanchis-Segura et al., 2018) than those entering non-STEM majors, whereas the opposite seems to be true for men.

However, gender-related stereotypes do not only act as gate-keepers that limit the access of many women to engineering and other STEM majors, they also hinder the achievement and persistence of those enrolled in them. These women face a highly-stereotyped environment in which they may constantly feel at risk of being stereotypically judged and their abilities and “belongingness” are permanently under question (Marra et al., 2009; Kim et al., 2018; Casad et al., 2019).

Fears about confirming stereotypes are generically called *stereotype threat* (Steele and Aronson, 1995; Steele, 1997). Experimental research has shown that, when explicit or implicitly threatened, stereotype-targeted individuals may exhibit reduced performance in stereotyped tasks and activities, including those related to math (Nguyen and Ryan, 2008; Picho et al., 2013; Flore and Wicherts, 2015). Stereotype threat can also reduce self-efficacy, interest, and sense of belonging in the stereotyped domain while increasing negative thoughts and anxiety (Lewis and Sekaquaptewa, 2016; Spencer et al., 2016). According to the stereotype threat theory (STT; Steele, 1997; Inzlicht and Schmader, 2011), stereotype threats can be triggered by a variety of environmental and interpersonal cues, most of which are frequently encountered in STEM academic contexts (e.g., numerical gender imbalance; for a review, see Murphy and Taylor, 2012). STT also posits that both the chances of experiencing a threat and its consequences are enhanced in individuals who are highly identified with the domain being tested, as is the case of math in female STEM students (Aronson et al., 1999; Pronin et al., 2004). Therefore, women enrolled in STEM studies are likely to experience diverse and repeated stereotype threat episodes that can potentially act as proximal contextual barriers and weaken their intentions of persisting in this field (Deemer et al., 2014; Cadaret et al., 2017; Lin and Deemer, 2021).

However, not all studies have found these stereotypes’ effects and it has been suggested that the consequences of stereotype threat can be dependent on interactions with personal factors (Stoet and Geary, 2012; Flore and Wicherts, 2015; Flore et al., 2018), hence affecting some but not all the members of the negatively stereotyped groups (e.g., women). Unfortunately, stereotype threat effects are better known than their mediators and moderators, hence limiting the chances of theoretically well-grounded interventions (Pennington et al., 2016). In this regard, only two experimental studies have assessed the possible moderating role of internalized gender stereotypes in the effects of stereotype threat on math self-efficacy (Good et al., 2008; Franceschini et al., 2014), and none of them evaluated motivational/persistence outcomes. Similarly, we are not aware of any previous experimental study assessing whether stereotype threat affects the persistence of STEM students in math-related activities through self-efficacy changes, and currently supporting evidence comes from field studies in which stereotype threat was not directly evaluated but self-reported (Deemer et al., 2014; Lin and Deemer, 2021). These voids motivated the present study.

### 1.3. Overview of the present study

The present study is developed within the frameworks provided by SCCT and STT, and was specifically designed to provide experimental evidence about the recently proposed role of stereotype threat as a possible proximal contextual barrier to women enrolled in STEM studies (Deemer et al., 2014; Cadaret et al., 2017). Specifically, we aimed to examine how experimentally activated stereotypes affect math self-efficacy, performance, and persistence of female and male engineering students in an effortful math task. Moreover, we also tested whether these effects were moderated by the strength and direction of the implicit gender-science stereotypes held by the participants before facing this task.

To pursue this goal, female and male engineering students were randomly assigned to one of two experimental conditions (control vs. stereotype threat activation), and the performance of these four experimental groups in a modified version of the Math Effort Task (Engle-Friedman et al., 2003) was compared. This task (hereafter referred to as M-MET) allowed us to obtain overall M-MET scores, but also separate measures of their principal components (total number of trials completed, percent of correctly solved problems, and level of difficulty chosen in each trial). These four dependent variables were interpreted as indexes of task performance, persistence, arithmetic accuracy, and self-confidence, respectively. In this last regard, it is worth noting that in the present study, overall math self-efficacy perceptions were also assessed by self-report before testing, hence obtaining two measures related to this construct that differed in their level of specificity (“overall” vs. task-specific and referred to as “math self-concept” and “in-task self-confidence”) and that, according to previous proposals (Pajares and Miller, 1994; Pajares, 1996), are expected to be less/more predictive of math performance and persistence, respectively.

As already mentioned, a second major goal of the present study was to assess to what extent the effects of the experimentally promoted threat were dependent on the participants’ “pre-testing stereotypes.” We use this term to refer to the stereotypical gender-science associations harbored by the participants before M-MET testing. These associations were evaluated with the implicit association test (IAT; Greenwald et al., 1998; Nosek et al., 2002a,b) and, therefore, the obtained estimates were independent of the participants’ ability and willingness to report them. These associations were expected to moderate M-MET performance in the threat, but not in the control, condition.

## 2. Materials and methods

The study was approved by the Ethics Standards Committees of the Universitat Jaume I.

### 2.1. Participants

A total of 340 undergraduate engineering students from the Universitat Jaume I (Castelló, Spain) and Universitat Politècnica de València (Valencia, Spain) who volunteered in response to an invitation email. The invitation email omitted any reference to gender or gender stereotypes and presented the current study as designed to “*evaluate some competences and intellectual abilities required to succeed in engineering*.”

The final recruited sample had an approximately similar proportion of self-reported males (175) and females (165). Female and male participants were randomly assigned to the control (Con) or the stereotype activation (StA) experimental conditions, resulting in four similarly sized groups that did not differ in age (Table 1).

**Table 1:** Demographic characteristics of the four participants’ groups.

	*N*	Median age	IQR age
M-Con	87	20	3
M-StA	88	20.5	3
F-Con	82	20	2.75
F-StA	83	20	2.5

The table reports the size of each experimental group and its median age. As reported in the main text, a two-way (gender x experimental condition) quantile-based ANOVA comparing the age medians revealed that the groups did not differ in age. IQR, Interquartile range; M-Con, males assigned to the control experimental condition; M-StA, males assigned to the stereotype activation condition; F-Con, females assigned to the control experimental condition; F-StA, females assigned to the stereotype activation condition.

All the participants signed informed consents, and their collaboration was rewarded with €20.

### 2.2. General procedure

The study was carried out across 18 different experimental sessions, each of them involving 15–20 participants with similar proportions of females and males. Two researchers greeted the participants to the laboratory and randomly assigned them to an individual desk equipped with a personal computer. After that, participants gave their informed consent and filled out a demographic data form. Finally, they sequentially completed the programmed tasks at the pace indicated by the leading researcher.

In order of presentation, the tasks and measures included in this study were:

- *Perceived competence/self-concept in specific academic domains*: Subjects were asked to rate themselves in four academic domains (math, physics, chemistry, and coding) by answering the question “*How good you are in*…?” on a 10-point visual scale. This kind of question has been ordinarily used to assess overall self-efficacy beliefs (see Wigfield and Eccles, 2000) and, in the present study, it was included to obtain a measure of each participant’s math self-concept (that is, his/her overall confidence in mathematics; see Parsons et al., 2009, 2011). Self-concepts in other academic domains were not of primary interest to the present study and their measurement was included to conceal the goals of the present study from the participants.
- *Implicit association test* (IAT): The Implicit Association Test (Greenwald et al., 1998) is built upon the observation that responding to information perceived as congruent is faster than responding to information perceived as incongruent. More specifically, the IAT measures the strength of the association between concepts and attributes by calculating the reaction time difference between stereotypical non-stereotypical concept-attribute pairs (see below), and it has been widely used to assess several implicit stereotypic associations (Greenwald et al., 1998; Nosek et al., 2002a,b). In this study, the well-validated “Gender-Science” IAT was used to measure the stereotypic “males-science” and “females-humanities” association (Nosek et al., 2002a,b; Smyth and Nosek, 2015). However, to avoid biasing the participants’ responses, this task was presented to the participants as measuring two cognitive abilities: “perceptual speed” and “concepts categorization.”

The “Gender-Science” IAT was implemented using a freely available script at the Millisecond Test Library,^1^ which we had previously adapted and translated into Spanish (see Sanchis-Segura et al., 2018). This script automatically counterbalances the order presentation of the test blocks containing stereotype-consistent trials and the blocks containing stereotype inconsistent trials, and it also automatically calculates the so-called IAT *D*-scores (Greenwald et al., 2003). The *D*-scores quantify the strength of the implicit association and are standardized deviation scores that range from +2 to −2 and whose interpretation is similar to that of the Cohen’s *d* statistic. Following the general convention, the IAT protocol used herein was arranged to provide positive *D* values for stereotype-consistent associations (e.g., “science = male/humanities = female”) and negative D values for stereotype-inconsistent associations.

- *Distraction task:* The Effort Expenditure for Rewards Task (EEfRT; Treadway et al., 2009) is a long (23 min) task that requires participants to select between “easy” and “hard” trials based on the probability of gaining different amounts of points by repeatedly and quickly pressing the spacebar a small/larger number of times, respectively. In this way, the EEfRT provides a measure of effort-based decision-making processes (e.g., Treadway et al., 2012). In the present study, the EEfRT was used as a mere distractor to temporally and mentally disengage the task assessing pre-existing gender-related stereotypes (IAT) from the task aimed to reactivate them (“*Reading comprehension task,”* see below). The EEfRT was implemented using the freely available script at the Millisecond Test Library.^2^ Data from 25 individuals were lost and those of the rest of the participants were not further used.
- “*Reading comprehension task”:* This task had two phases (reading and comprehension testing) and was used to inadvertently promote the activation of gender-related stereotypes through the reading of a text.

Thus, all participants were instructed to carefully read a newspaper article that was about to be displayed on their computer screens. Participants were also informed that later on they would have to answer seven “yes/ no” questions designed to evaluate their understanding of the article’s contents. Participants could freely navigate through the two-page document for 10 min (reading phase). However, the material presented to the participants of the CON and StA groups differed. Thus, the text presented to the participants of the CON group was a real press article about the use of smartphones in primary school activities that had been published on the webpage of a national newspaper. Conversely, the text presented to the StA group had exactly the same layout as the original article, but its contents (text and illustrations) were replaced by others containing made-up psychological and neuroimaging “findings” supposedly proving that men (and their brains) are more “mathematical” and less “verbal” whereas women (and their brains) are more “verbal” and less “mathematical.” Once the pre-established reading time had elapsed, participants were instructed to start the testing phase and answer the seven upcoming questions by pressing the “b” key (masked with a green tag) or the “n” key (masked with a red tag) to provide “yes” or “no” answers, respectively. To avoid including non-complying participants, an exclusion criterion (i.e., providing two or more incorrect answers in the reading comprehension test) was set beforehand. However, no subjects needed to be actually excluded for this reason. The scores obtained in this task were not used further.

- *Modified math effort task (M-MET)*: The math effort task (MET) was originally developed by Engle-Friedman et al. (2003), and consists of 100 independent addition problems conducted after five practice trials (one per difficulty level, see below) at which performance feedback (“correct”/“false”) is provided. Each problem consists of 4 numbers that are flashed onto the screen one by one for 800 milliseconds and participants are asked to add the numbers in their heads. In each trial, the numbers for each problem are selected randomly but differ according to the participants’ self-selected level of difficulty, so the presented numbers are in the range of 0–2 (Level 1), 2–8 (Level 2), 6–13 (Level 3), 10–25 (Level 4), and 12–35 (Level 5). Once the last number is erased, a textbox is provided to collect the participants’ responses in the following 15 s, and the next trial starts without offering any performance feedback information.

In the present study, the MET was implemented through the freely available script of the Millisecond test library.^3^ However, we modified the task in order to allow participants to choose the difficulty of each problem (instead of each 5 consecutive trials) and to have the opportunity to voluntarily abandon the task after finishing each trial. Thus, after these modifications, this task provided us with 4 different dependent variables:

1. *M-MET global scores*: As in the original MET, M-MET global scores summarize the individuals’ performance in this task and are calculated through the formula: SCORE = Σ[(correct solutions to level 5 problems * 1) + (correct solutions to level 4 problems * 0.8) + (correct solutions to level 3 problems * 0.6) + (correct solutions to level 2 problems * 0.4) + (correct solutions to level 1 problems * 0.2)].
2. *Number of completed trials:* Unlike the original MET, this variable was under the participants’ control in our task and provided an individual measure of their motivation or willingness to persist in a math task that requires sustained effort. Note that this measure is calculated in a manner that is not affected by the participants’ chosen difficulty and their mental calculation abilities, hence isolating this motivational aspect.
3. *Chosen difficulty*: As mentioned in the introduction section, chosen difficulty was interpreted as a measure of in-task self-confidence. This was first assessed at the group level by comparing the relative frequency of trials completed for each selected difficulty level in each group. Of higher interest, the average difficulty level chosen by each participant (individual average chosen difficulty; I-ACD) was also calculated and these individual scores were employed to conduct more detailed group-based comparisons but also correlational analyses. Note that because they are calculated as averages or relative frequencies across all completed trials, the values of these two measures are not dependent on the number of trials completed or the participants’ arithmetic accuracy.
4. *Arithmetic accuracy:* Accuracy, operationalized as the percent of correctly solved problems (over the number of problems attempted) was used as a measure of sustained arithmetic performance.

### 2.3. Statistical analyses

All statistical analyses were conducted in R (R Core Team, 2019). Statistical analyses focused on description and effect size estimation, as much as on testing statistical significance (Wasserstein and Lazar, 2016), and employed robust, non-parametric methods that simultaneously compared several location measures of the groups’ distributions without making any pre-assumption about their shape or variance (Wilcox, 2016; Rousselet et al., 2017). In all analyses involving multiple comparisons, value of *p*s were adjusted with the Hochberg method and only those adjusted values are reported.

Thus, in the present study, between-group comparisons were conducted with robust equivalents of classic two-way (gender x experimental condition) ANOVAs comparing the quartile values (Q25, Q50, and Q75) instead of just the groups’ means. The omnibus tests of these quantile-ANOVAs (qANOVAs) were obtained with the *q2by2* function and, when appropriate, the *Qmcp* function was employed to implement dyadic post-hoc comparisons at the quantile(s) of interest (see Wilcox, 2016; Rousselet et al., 2017). These two functions use the robust Harrell-Davis quantile estimator and a percentile bootstrap approach (2000 iterations) for effects and value of *p*s estimation and, when multiple comparisons are performed, control the probability of Type I errors with the Hochberg’s method. In these comparisons, the unstandardized difference between the estimated quantile values (denoted as d^ ) and its corresponding 95% confidence intervals (calculated by the same percentile bootstrap method previously referred to) were employed to estimate the size of the observed effects.

Moreover, complementary comparisons involving the entire groups’ distributions were conducted using the two-samples Kolmogorov-Smirnoff (K-S) and the Cliff’s delta tests (Wilcox, 2016). The D statistic of the K-S test and the probability of superiority (PS) were employed as measures of effect size in these comparisons. The K-S D informs of the absolute value of the largest difference between the empirical cumulative distribution functions of the compared groups, whereas the PS is a probabilistic effect size index that denotes the probability that a randomly sampled member of group A will have a higher score than the score attained by a randomly sampled member of group B (Grissom and Kim, 2012).

On the other hand, some of the dependent variables considered in the present study allowed or required additional or alternative analytical approaches. More specifically:

- Given that the number of trials in the M-MET is under the participants’ control but limited to a maximum value of 100, it is a time-to-event, right-censored variable that requires a specific statistical treatment (Lemeshow et al., 2011). Thus, to analyze possible group differences in the number of trials completed before abandoning or completing the M-MET, survival curves were calculated using the Kaplan–Meier’s method. These curves were compared with the Long-Rank test followed by pairwise comparisons. All these analyses were conducted using different functions provided by the *survival* package (Therneau, 2022).
- Between group differences in chosen difficulty were assessed using the K-S test and the qANOVA methods previously described on the I-ACD scores, but also through contingency tables relating the frequency at which each difficulty level was chosen by the members of each group. The association between these two categorical variables was assessed with the Pearson’s chi-squared test and the Pearsons’ residuals were inspected to identify which cells contributed the most to the overall test result (Sharpe, 2019). These analyses were conducted with the functions provided by the *vcd* package (Zeileis et al., 2007).
- Group differences in arithmetic performance were first assessed by applying the K-S test and the qANOVA methods previously described to the arithmetic accuracy scores. Given that accuracy scores are independent of the number of completed trials but not of the participants’ chosen difficulty (i.e., arithmetic accuracy is expected to be higher in easier than in more difficult problems), additional between-group comparisons were conducted with a robust ANCOVA-like method. More specifically, the *ancmg1* function (see Wilcox, 2016) was employed to compare the groups’ accuracy medians after removing the effects of chosen difficulty by treating I-ACD scores as a covariate of no interest.

In addition to these comparisons between groups, transversal analyses across genders and analyses assessing individual differences within each gender category were conducted.

Regarding the former, IAT D-scores were first transformed into the “IAT-influence” scores (see Sanchis-Segura et al., 2018) and then the relationships between M-MET performance (global and subcomponent scores), math self-concept, and the obtained “IAT-influence” scores of all (male and female) participants subjected to each experimental condition were jointly assessed with correlational methods. This transformation: (1) is needed when jointly analyzing IAT D-scores of female and male participants because the same score has different implications -and it is expected to have opposite consequences in task performance- for the members of each gender; (2) just involves multiplying males’ IAT scores by 1 and those of females by −1 and, therefore, does not change the strength of the IAT-measured association nor those of its possible correlations with other variables; and, (3) enriches the interpretation of these correlations, as they quantify the strength and sign of the expected impact (“influence”) of the IAT-measured association over other variables and not just their co-variation. These IAT “influence” scores were first employed in robust regression-based moderation analyses, which were carried out using the *lmrob* function of the *robustbase* package for R and that were aimed to assess if -as hypothesized- the participants’ pre-testing stereotypes did only affect M-MET performance in the participants subjected to the StA condition. Given the results obtained in these analyses, between variables’ relationships were separately explored in each experimental condition using two complementary strategies. First, the strength of the zero-order association between each pair of variables was quantified using Spearman’s rho correlation index. *The values of the obtained correlations were compared using the twopcor and twohc4cor functions* (Wilcox, 2016). Second, LASSO-regularized partial correlation networks were built to describe the whole pattern of direct associations between these variables and unbiasedly estimate their strength (Epskamp et al., 2012; Epskamp and Fried, 2018). More specifically, separate partial Spearman correlation networks for each experimental condition were first estimated with the *estimateNetwork* function (default = “EBICglasso,” gamma = 0.5) of the *bootnet* package (Epskamp et al., 2018) and subsequently compared with the *NCT* function of the *NetworkComparisonTest* package (Van Borkulo et al., 2022). To avoid the distortions derived from conditioning on a *common effect* (Pearl, 2000; Epskamp and Fried, 2018), overall M-MET scores (which are arithmetic composites of the number of completed trials, accuracy, and I-ACD scores) were not included in the construction of these networks.

On the other hand, we hypothesized that, if the opposite effects of stereotype threat on the M-MET performance of males and females are influenced by pre-existing implicit gender-science associations, changes in M-MET performance should be solely observed to be more prominent in those individuals of each gender holding stereotypical gender-science implicit associations (i.e., IAT D-scores >0). Conversely, stereotype threat effects should probably be minimized, absent, or even reversed in those members of the M-StA and F-StA groups lacking such stereotypes or harboring counter-stereotypic associations (i.e., IAT D-scores ≤ 0).To test this hypothesis, within-gender comparisons between participants receiving distinct (positive vs. negative) “influences” from their own implicit “gender-science” associations (i.e., IAT “influence” scores higher or lower than 0, respectively) were conducted. More specifically, the *qcomhd* function (Wilcox, 2016) was employed to compare each of these two subgroups of females and males exposed to the StA condition between them and also with their respective control groups regarding M-MET, I-ACD, and accuracy scores. To perform the same within-gender comparisons on the number of completed trials, the previously described Kaplan–Meier’s method was employed.

## 3. Results

Table 1 summarizes the composition of the experimental groups recruited for the present study. A two-way (gender x experimental condition) qANOVA comparing the age medians confirmed that participants’ age did not differ between groups (gender, *p* = 0.118; experimental condition, *p* = 0.605; gender x experimental condition, *p* = 0.967).

### 3.1. Self-concept of males and females in different academic domains

A series of two-way (gender x experimental condition) qANOVAs comparing the participants’ overall self-efficacy beliefs yielded significant effects of gender in some, but not all, of the evaluated academic domains (Table 2). More specifically, males rated themselves higher than females in coding (Q25: *p* < 0.006, d^ =1.31 [0.38, 1.96]; Q50: *p* < 0.001, d^ =1.32 [0.51, 2.00]; Q75: *p* < 0.001, d^ =1.54 [0.77, 2.42]) and to a lesser extent in physics (Q25: *p* = 0.05, d^ =0.81 [−0.01, 1.77]; Q50: *p* = 0.027, d^ =0.35 [0.03, 1.06]; Q75: *p* = 0.144, d^ =0.81 [−0.02, 0.65]). Conversely, females and males did not significantly differ in their perceived self-concept in chemistry or math (see Table 2 for details).

**Table 2:** Self-concept in different academic domains in the four participants’ groups.

	GROUP	Q25	Q5	Q75
CODING	M-Con	5.22	7.22	8.90
	M-StA	4.52	6.49	8.03
	F-Con	3.22	5.74	7.13
	F-StA	3.87	5.46	6.92
		**Gender, *p* = 0.006**	**Gender, *p* < 0.001**	**Gender, *p* < 0.001**
		Condition, *p* = 0.812	Condition, *p* = 0.280	Condition, *p* = 280
		Interaction, *p* = 0.357	Interaction, *p* = 0.378	Interaction, *p* = 0.378
PHYSICS	M-Con	5.89	7.05	8.00
	M-StA	5.60	7.01	8.00
	F-Con	4.93	6.50	7.87
	F-StA	4.85	6.71	7.86
		Gender, *p =* 0.050	**Gender, *p* < 0.027**	Gender, *p =* 0.144
		Condition, *p* = 0.931	Condition, *p* = 0.931	Condition, *p* = 0.931
		Interaction, *p* = 0.946	Interaction, *p* = 0.946	Interaction, *p* = 0.946
CHEMISTRY	M-Con	3.33	5.09	7.09
	M-StA	3.47	5.40	7.41
	F-Con	4.27	6.23	7.89
	F-StA	3.27	5.09	7.43
		Gender, *p =* 0.351	Gender, *p =* 0.351	Gender, *p =* 0.351
		Condition, *p* = 0.720	Condition, *p* = 0.720	Condition, *p* = 0.810
		Interaction, *p* = 0.249	Interaction, *p* = 0.249	Interaction, *p* = 0.237
MATH	M-Con	6.19	7.17	8.34
	M-StA	6.01	7.33	8.32
	F-Con	6.12	7.67	8.66
	F-StA	6.01	7.22	8.34
		Gender, *p =* 0.945	Gender, *p =* 0.945	Gender, *p =* 0.945
		Condition, *p* = 0.691	Condition, *p* = 0.691	Condition, *p* = 0.691
		Interaction, *p* = 0.964	Interaction, *p* = 0.964	Interaction, *p* = 0.964

Displayed group scores correspond to the empirical quartile values. Statistical significance was tested using two-way (gender x condition) quantile-based ANOVAs and the reported *value of p*s correspond to the main factors and interactions of these tests. All value of *p*s were adjusted for multiple comparisons using the Hochberg method. Statistically significant effects are highlighted in bold. M-Con, males assigned to the control experimental condition; M-StA, males assigned to the stereotype activation condition; F-Con, females assigned to the control experimental condition; F-StA, females assigned to the stereotype activation condition.

When the entire distributions of these self-concept scores were compared through a series of Kolmogorov-Smirnoff tests, significant gender-based differences were also found in the participants’ self-concept in coding and physics (K-S D_coding_ = 0.25, *p* < 0.001; K-S D_pyisics_ = 0.16, *p* = 0.027) but not in chemistry or math (K-S D_chemistry_ = 0.07, *p* = 0.678; K-S D_math_ = 0.03, *p* > 0.999). Accordingly, the probability that a randomly picked male would judge himself as more competent than a randomly picked female in coding (PS_Males_ = 0.63 [0.57, 0.68]) or physics (PS_Males_ = 0.58 [0.52, 0.64]) was significantly higher than for the reverse comparisons (Cliff’s delta = 0.26 and 0.16, *p* < 0.001 and *p* = 0.002, respectively).

Taken together, these results indicate that although males rated themselves higher than females in some academic domains, females and males perceived themselves as similarly competent in math. Moreover, it should be highlighted that math self-concept scores did not differ between the male nor the female participants randomly assigned to the control and StA conditions, hence ruling out any possible contribution of this variable to other possible differences between the M-Con/ M-StA and the F-Con/ F-StA groups.

### 3.2. “Gender-science” implicit association test

**Figure 1:**
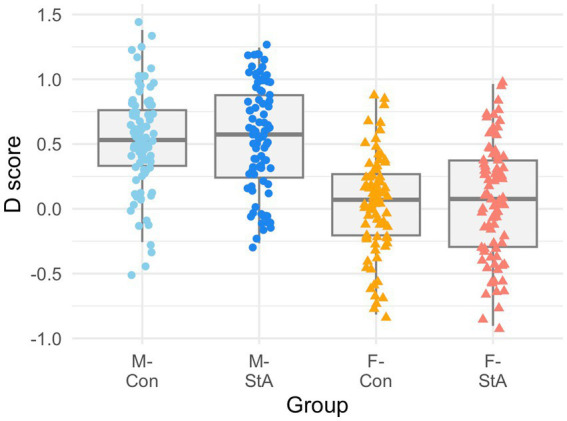
Participants’ scores in the “Gender-Science” IAT. The graph depicts the groups’ distributions (boxplot) and the individual D-scores of the participants in the “Gender-Science” IAT. *D*-scores quantify the strength of the implicit association and their interpretation is similar to Cohen’s *d*. Following the general convention, positive *D* values denote stereotype-consistent associations (i.e., “science = male/humanities = female”) and negative D values for stereotype-inconsistent (i.e., “science = female/humanities = male”) associations. Statistically significant, gender-based differences (males > females) were found at the three quartile values (see main text for details). IAT, implicit association test; M-Con, males assigned to the control experimental condition; M-StA, males assigned to the stereotypes’ reactivation condition; F-Con, females assigned to the control experimental condition; F-StA, females assigned to the stereotypes’ reactivation condition.

Figure 1 depicts the participants’ D-scores in the “Gender-Science” IAT. A two-way qANOVA yielded a statistically significant effect of the gender factor (*p* < 0.001 in all three quartiles), but not of the experimental condition factor (Q25: *p* = 0.392; Q50: *p* = 0.392; Q75: *p* = 0.093), nor of the between-factors’ interaction (*p* > 0.99 in all three quartiles). Gender-based differences were similar in size at the three quartiles ( d^ =0.50 [0.34, 0.64], d^ =0.46 [0.36, 0.55], d^ =0.49 [0.38, 0.59], respectively).

In the same line, a Kolmogorov-Smirnoff test confirmed that the distribution of the “Gender-Science” IAT scores differed between males and females (K-S D = 0.48, *p* < 0.001). Thus, the probability that a randomly picked male would obtain a higher D-score in this IAT (PS_Males_ = 0.79 [0.74, 0.84]) was substantially higher than for the reverse comparison (PS_Males_ = 0.21 [0.16, 0.26]; Cliff delta = 0.59, *p* < 0.001).

Therefore, as it could be expected from previous studies (e.g., Smyth and Nosek, 2015), we observed that male engineering students exhibit a stronger stereotypical “male-science/female-humanities” association than female engineering students. In fact, this association was harbored by the large majority of these male students (i.e., 87.43% of them had IAT D-scores >0) but only by around half (58.19%) of the female students. On the other hand, it should be noted that, as also observed for self-efficacy scores, neither the male nor the female participants randomly assigned to their respective Con and StA groups differed in their “Gender-Science” IAT. Therefore, any later difference between the M-Con/ M-StA and the F-Con/ F-StA groups should be attributed to the effects of stereotype activation and not to any pre-existing differences between these groups.

### 3.3. Modified math effort task (M-MET)

#### 3.3.1. Global M-MET scores

**Figure 2:**
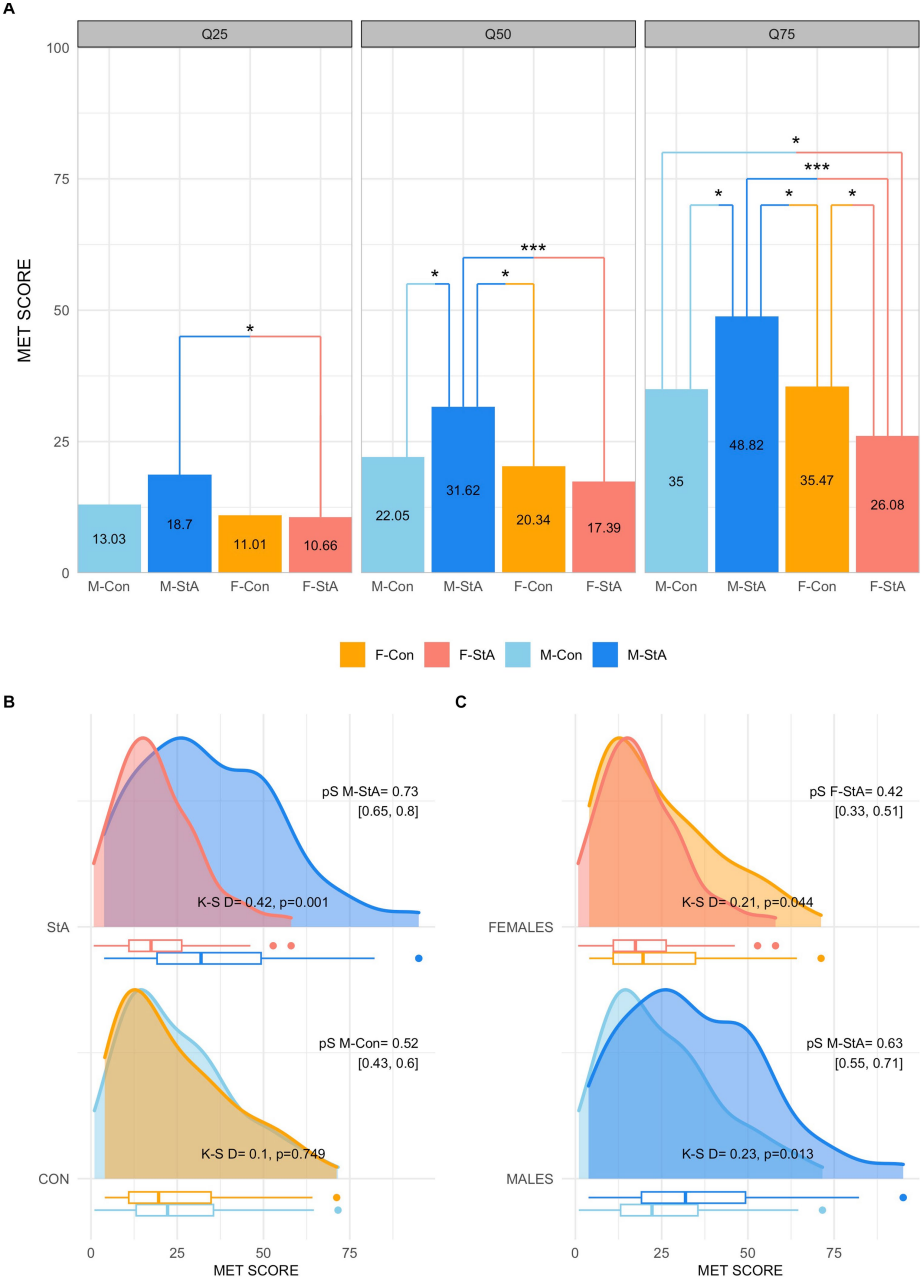
Participants’ scores in the overall M-MET (modified math effort task). Panel **(A)** shows the values of the overall M-MET scores of each group at each quartile (Q25, Q50, Q75), as well as the results of their comparisons (*p < 0.05, **p < 0.01, ***p < 0.001 after correction for multiple comparisons with the Hochberg method). Panel **(B)** depicts the whole distributions of the M-MET scores of the groups subjected to stereotypes activation (top) and control condition (bottom). Panel **(C)** illustrates the same distributions but compares them based on the gender factor (females, top; males, bottom). M-Con, males assigned to the control experimental condition; M-StA, males assigned to the stereotypes’ reactivation condition; F-Con, females assigned to the control experimental condition; F-StA, females assigned to the stereotypes’ reactivation condition; Con, control condition; StA, stereotypes’ reactivation condition; K-S, Kogolmorov-Smirnoff test; PS, probability of superiority.

A two-way (gender x experimental condition) qANOVA comparing the participants’ M-MET yielded statistically significant effects of the gender factor (*p* < 0.005 in the three quartile values) and of their interactions with the experimental condition factor (Q25: *p* = 0.043; Q50: *p* = 0.012; Q75: *p* < 0.001). As illustrated in panel A of Figure 2, post-hoc dyadic comparisons revealed that: 1) At Q25, statistically significant differences were solely observed between the F-StA and the M-StA groups; 2) At Q50, the M-StA group exhibited larger M-MET scores than the F-StA but also than the M-Con and the F-Con groups, which were not significantly different between them nor the F-StA group; and 3) At Q75, the M-StA exhibited larger M-MET scores than all the other groups, the F-StA exhibited smaller M-MET scores than all other groups whereas the M-Con and F-Con did not significantly differ between each other. The details of these comparisons can be found in Table 3. These results suggest that the stereotype activation produced opposite effects in females and males, hence promoting gender-based differences in M-MET scores in the StA condition that were not observed in the control condition. Note that these effects seemed to be more prominent for males than for females and for higher (Q75) than for lower (Q25) M-MET scores (Table 3).

**Table 3:** Between-group differences in overall M-MET scores.

Quantile	Comparison	*p* value	d^	95% CI
Q25	M-StA vs. M-Con	0.170	5.67	[1.40, 13.46]
	M-StA vs. F-Con	0.053	7.7	[−0.04, 15.27]
	**M-StA vs. F-StA**	**0.015**	**8.04**	**[1.12, 15.60]**
	F-StA vs. M-Con	0.228	−2.37	[−6.35, 2.52]
	F-StA vs. F-Con	0.814	−0.34	[−5.73, 4.08]
	M-Con vs. F-Con	0.679	2.03	[−3.81, 6.08]
Q50	**M-StA vs. M-Con**	**0.014**	**9.58**	**[0.823, 20.82]**
	**M-StA vs. F-Con**	**0.012**	**11.29**	**[1.80, 21.40]**
	**M-StA vs. F-StA**	**<0.001**	**14.23**	**[7.32, 24.29]**
	F-StA vs. M-Con	0.270	−4.65	[−11.95, −2.11]
	F-StA vs. F-Con	0.428	−2.95	[−11.79, 3.26]
	M-Con vs. F-Con	0.652	1.71	[−8.46, 10.20]
Q75	**M-StA vs. M-Con**	**0.015**	**13.82**	**[1.69, 21.53]**
	**M-StA vs. F-Con**	**0.015**	**13.35**	**[1.15, 22.34]**
	**M-StA vs. F-StA**	**<0.001**	**22.74**	**[13.37, 30.28]**
	**F-StA vs. M-Con**	**0.015**	**−8.92**	**[− 19.55, −1.16]**
	**F-StA vs. F-Con**	**0.017**	**−9.39**	**[−20.50, −0.05]**
	M-Con vs. F-Con	0.932	−0.47	[−11.52, 11.17]

The table reports the outcome (*p*-value, quantile difference, and its corresponding 95% CI) of follow-up post-hoc comparisons conducted after the identification of statistically significant gender x group interactions in quantile-based ANOVAs comparing overall M-MET scores (see main text). Robust post-hoc comparisons were carried out with the *Qmcp* function, and those reaching statistical significance after multiple comparisons correction with the Hochberg’s method are highlighted in bold. M-Con, males assigned to the control experimental condition; M-StA, males assigned to the stereotype activation condition; F-Con, females assigned to the control experimental condition; F-StA, females assigned to the stereotype activation condition; CI, confidence interval.

In agreement with these results, the M-MET score distributions of the M-Con and F-Con groups did not significantly differ between them (*D* = 0.1, *p* = 0.749; Figure 2A, bottom), but the M-MET score distributions of the M-StA and F-StA groups did (D = 0.42, *p* = 0.001; Figure 2A, top). Thus, the probability that a randomly selected individual of the M-StA group would obtain a higher M-MET score than that obtained by a randomly selected member of the F-StA group (PS M-StA = 0.73 [0.65, 0.80]) was significantly higher (Cliff’s delta = 0.46, *p* < 0.001) than that for the reverse comparison (PS F-StA = 0.27 [0.20, 0.35]), a difference that was not observed in the control condition (Cliff’s delta = 0.03, *p* = 0.710). As illustrated by panel B of the same figure, gender-based differences in the StA condition seem to stem from two statistically significant but opposite sign effects of stereotype activation in females and males. Thus, compared to the distributions of their respective control groups, the M-MET score distribution of the F-StA and M-StA were significantly shifted toward lower (*D* = 0.21, *p* = 0.044) and toward higher (*D* = 0.23, *p* = 0.013) values, respectively. Note that, as indicated by the obtained Cliff’s delta values, these effects appeared to be slightly more prominent in males (Cliff’s delta = 0.26, *p* = 0.003) than in females (Cliff’s delta = −0.16, *p* = 0.08), an effect that seems to align with the observation that the M-StA, but not the F-StA, differed from their respective control group at intermediate levels of M-MET performance (Q50, Table 3).

Taken together, these results confirm that the activation of stereotypes about a gender-based difference in math competence boosted the performance of males and, to a lower extent, also decreased that of females in an effortful, math-related task.

#### 3.3.2. How stereotype threat affects M-MET scores?

As described in the methods section, global M-MET scores depend on three separate components: The number of trials completed, the chosen difficulty level at each trial, and arithmetic accuracy (the proportion of correctly solved problems). Therefore, we investigated which of these components were affected by the induced stereotype threat and underlie the between-group differences of the M-MET scores described in Section 3.3.1.

##### 3.3.2.1. Does stereotype threat affect the number of trials completed?

**Figure 3:**
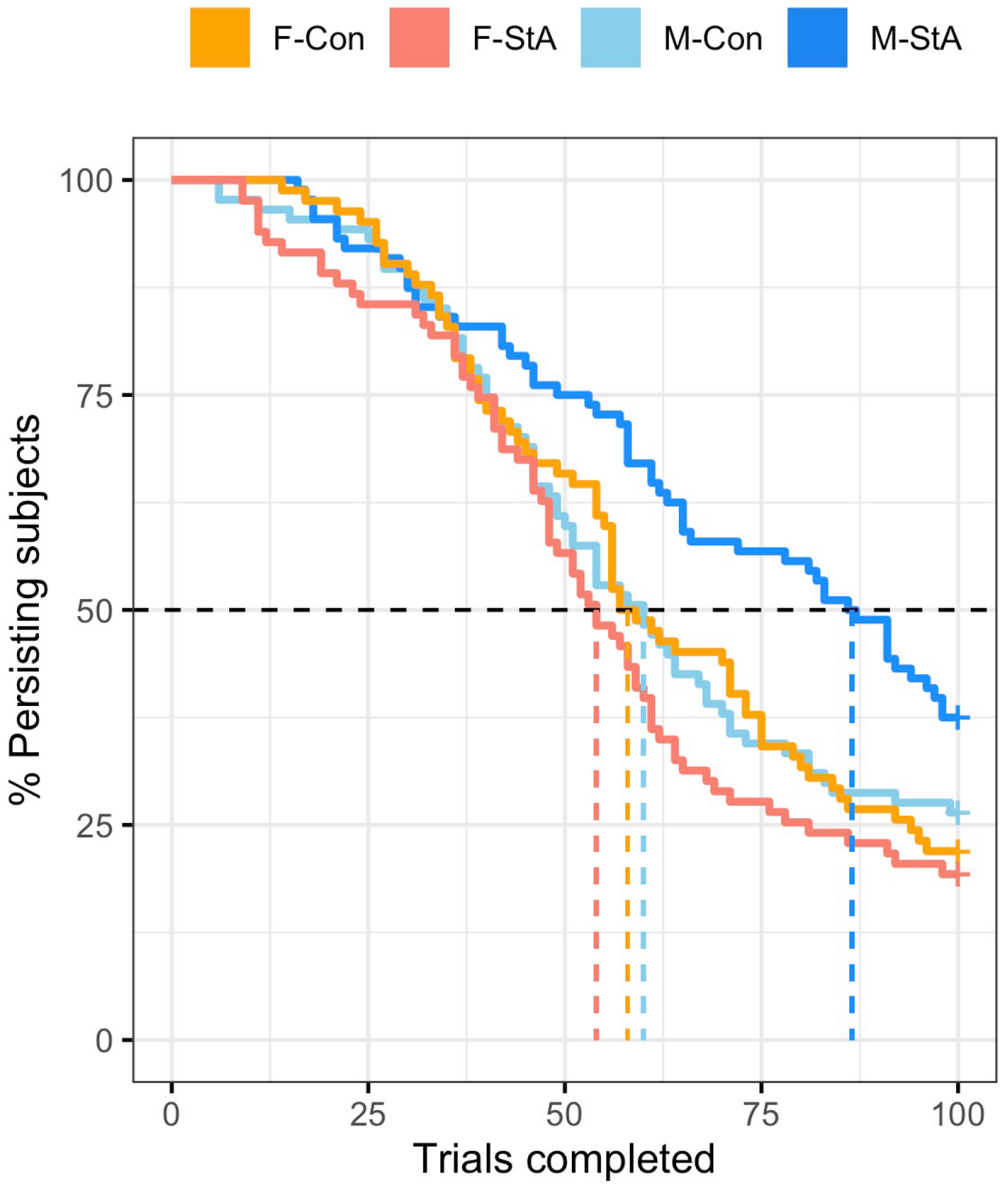
Participants’ persistence in the M-MET (modified math effort task). This figure depicts the percentage of subjects of each group that persisted in the task across trials. The colored stepped lines correspond to the Kaplan–Meier estimates of the survival curves of each group, whereas the dashed vertical lines indicate the median survival time in each group. M-Con, males assigned to the control experimental condition; M-StA, males assigned to the stereotypes’ reactivation condition; F-Con, females assigned to the control experimental condition; F-StA, females assigned to the stereotypes’ reactivation condition.

A Survival analysis (Kaplan–Meier) was used to determine whether there were between-group differences regarding the proportion of individuals persisting in the M-MET task across trials. As it can be readily observed from the survival distribution curves depicted in Figure 3, the members of the M-StA persisted longer in the M-MET than the members of all other groups. The same conclusion was obtained when analyzing the median survival times, which reported estimated values of 86.5, 60, 58, and 54 for the M-StA, M-Con, F-Con, and F-StA groups, respectively. Confirming these observations, a Long-Rank test yielded a significant group effect (*χ*^2^(3) = 12.1, *p* = 0.007) and post-hoc comparisons only yielded statistically significant differences between the survival curve of the M-Sta group and those of all other groups (M-StA vs. M-Con, *p =* 0.031; M-StA vs. F-Con, *p =* 0.011; M-StA vs. F-StA, *p <* 0.001). Taken together, these results suggest that stereotype activation selectively increased the persistence on the M-MET task in males.

##### 3.3.2.2. Does stereotype threat affect the chosen trials’ difficulty?

**Figure 4:**
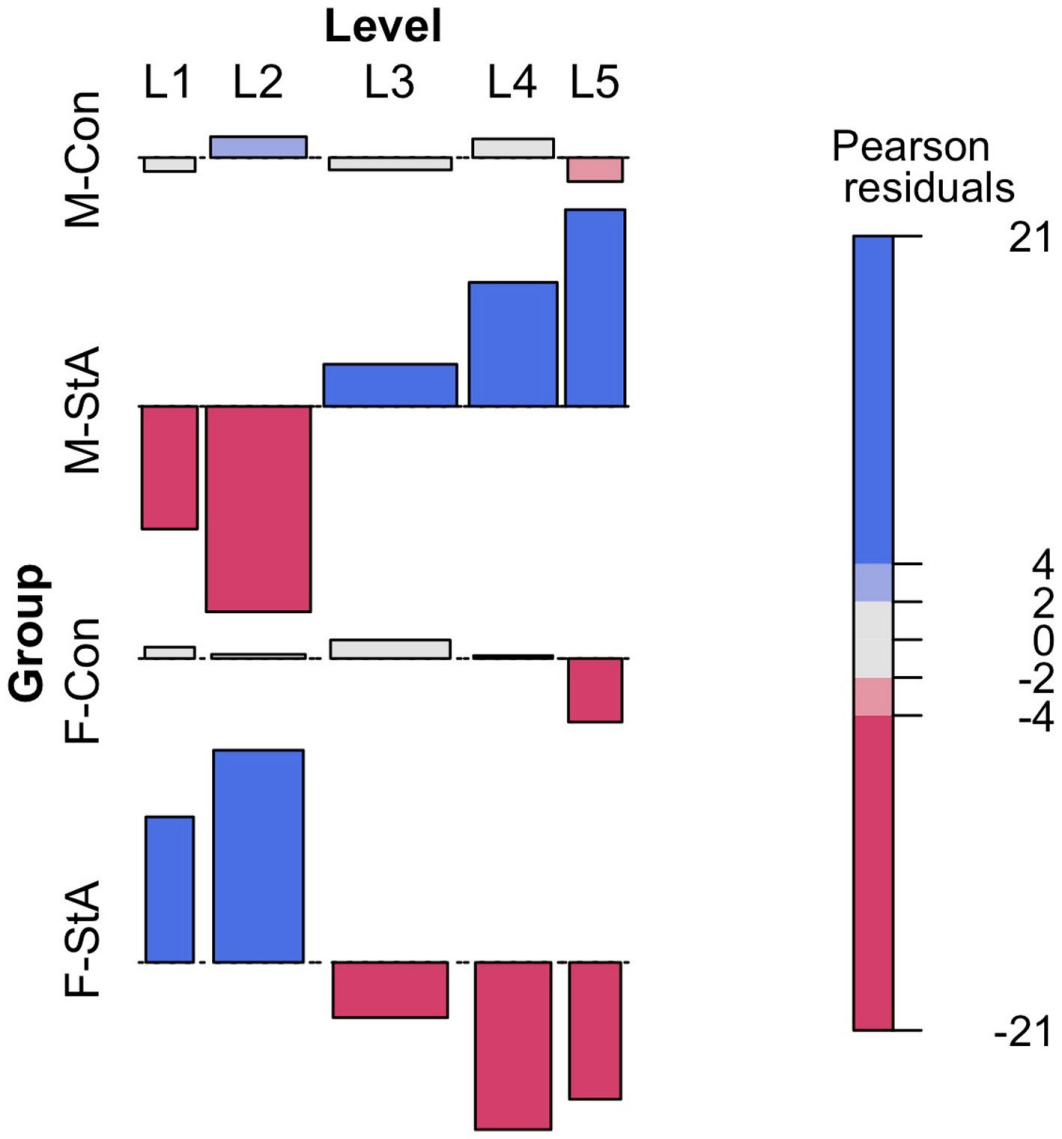
Participants’ selected difficulty in the M-MET (modified math effort task). The figure presents an association plot depicting the groups’ deviations from a theoretically homogeneous distribution of trials across difficulty levels. Thus, bars projecting above/below the horizontal baseline denote difficulty levels chosen more/less than theoretically expected, with the rectangle area being proportional to the difference between the expected and observed values (the height is proportional to the Pearson’s residual value and the width is proportional to the square root of the expected frequency). M-Con, males assigned to the control experimental condition; M-StA, males assigned to the stereotypes’ reactivation condition; F-Con, females assigned to the control experimental condition; F-StA, females assigned to the stereotypes’ reactivation condition.

A Pearson’s chi-square test (*χ*^2^(12) = 237.1, *p* < 0.001) showed that chosen difficulty level differed between groups and the analysis of the residuals (Figure 4) revealed that M-StA and F-StA groups were the ones showing a larger deviation from the chance-expected values. More specifically, the individuals of the M-StA group rarely opted for the least difficult levels (L1 and L2) and chose the most difficult ones (especially, L5) more than could be expected. Members of the F-StA exhibited the exact opposite pattern of choices, thus opting for the easiest problems and avoiding the most difficult ones (L5). Deviations from the expected values were also observed in the M-Con and F-Con groups, but they were much smaller in size and almost exclusively affecting the most difficult level (L5). These results suggest that stereotype threat promoted opposite effects in females and males, making them select less/more difficult problems, respectively.

**Figure 5:**
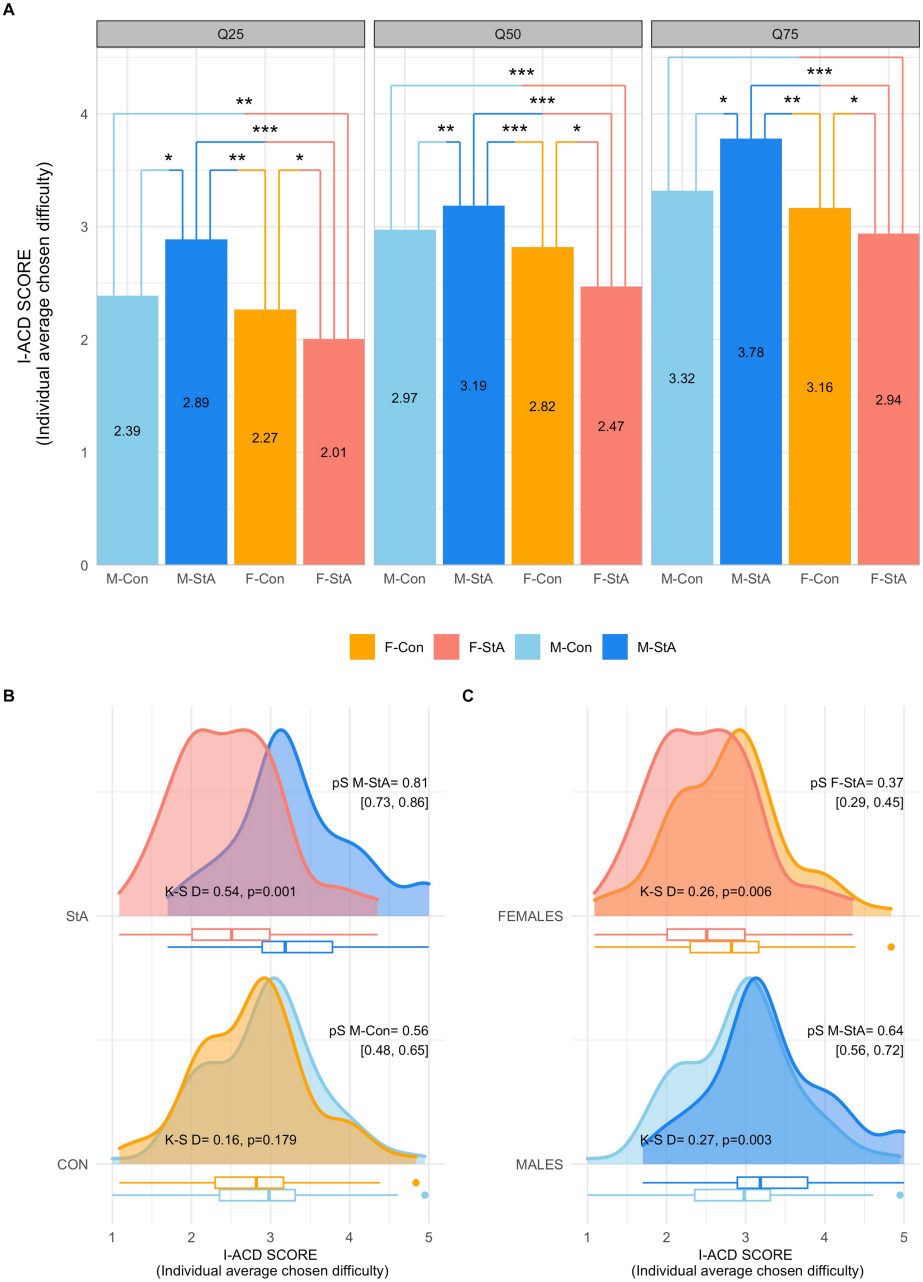
Participants’ scores in the I-ACD scores (individual average chosen difficulty) in the M-MET. Panel **(A)** shows the values of the I-ACD scores of each group at each quartile value (Q25, Q50, Q75), as well as the results of their comparisons (*p < 0.05, **p < 0.01, ***p < 0.001 after correction for multiple comparisons with the Hochberg method). Panel **(B)** depicts the whole distributions of the I-ACD scores of the groups subjected to stereotypes’ reactivation (top) and control condition (bottom). Panel **(C)** illustrates the same distributions but compares them based on the gender factor (females, top; males, bottom). M-Con, males assigned to the control experimental condition; M-StA, males assigned to the stereotypes’ reactivation condition; F-Con, females assigned to the control experimental condition; F-StA, females assigned to the stereotypes’ reactivation condition; Con, control condition; StA, stereotypes’ reactivation condition; K-S, Kogolmorov-Smirnoff test; PS, probability of superiority.

In the same line, a two-way qANOVA (gender × experimental condition) comparing the I-ACD scores among groups yielded statistically significant effects of the gender factor (*p* < 0.001) and of its interaction (*p* < 0.005) with the experimental condition factor in the three quartile values. As illustrated in panel A of Figure 5, these global effects were a result of a pattern of between group differences (M-StA > M-Con = F-Con > F-StA) that was repeated, with a similar size across the three quartile values (see Table 4 for details).

**Table 4:** Between-group differences in I-ACD scores.

Quantile	Comparison	*p* value	d^	95% CI
Q25	**M-StA vs. M-Con**	**0.019**	**0.50**	**[0.03, 0.84]**
	**M-StA vs. F-Con**	**0.002**	**0.62**	**[0.17, 0.91]**
	**M-StA vs. F-StA**	**<0.001**	**0.88**	**[0.50, 1.17]**
	**F-StA vs. M-Con**	**0.002**	**−0.38**	**[−0.84, −0.06]**
	**F-StA vs. F-Con**	**0.027**	**−0.26**	**[−0.62, −0.03]**
	M-Con vs. F-Con	0.414	0.12	[−0.29, 0.58]
Q50	**M-StA vs. M-Con**	**0.006**	**0.21**	**[0.04, 0.48]**
	**M-StA vs. F-Con**	**<0.001**	**0.37**	**[0.17, 0.65]**
	**M-StA vs. F-StA**	**<0.001**	**0.72**	**[0.47, 1.09]**
	**F-StA vs. M-Con**	**<0.001**	**−0.50**	**[−0.81, −0.23]**
	**F-StA vs. F-Con**	**0.012**	**−0.35**	**[−0.67, −0.02]**
	M-Con vs. F-Con	0.066	0.15	[−0.08, 0.44]
Q75	**M-StA vs. M-Con**	**0.032**	**0.46**	**[0.01, 0.89]**
	**M-StA vs. F-Con**	**0.006**	**0.61**	**[0.14, 1.03]**
	**M-StA vs. F-StA**	**<0.001**	**0.84**	**[0.41, 1.32]**
	**F-StA vs. M-Con**	**<0.001**	**−0.38**	**[−0.75, −0.13]**
	**F-StA vs. F-Con**	**0.032**	**−0.22**	**[−0.59, −0.02]**
	M-Con vs. F-Con	0.199	0.15	[−0.18, 0.48]

The table reports the outcome (*p*-value, quantile difference, and its corresponding 95% CI) of dyadic post-hoc comparisons conducted after the identification of statistically significant gender x group interactions in quantile-based ANOVAs comparing I-ACD scores. Robust post-hoc comparisons were carried out with the *Qmcp* function, and those reaching statistical significance after multiple comparisons correction with the Hochberg’s method are highlighted in bold. I-ACD, individual average chosen difficulty; M-Con, males assigned to the control experimental condition; M-StA, males assigned to the stereotype activation condition; F-Con, females assigned to the control experimental condition; F-StA, females assigned to the stereotype activation condition; CI, confidence interval.

These effects were further characterized by comparing the groups’ I-ACD score distributions. These distributions did not differ between males and females in the control condition (K-S D = 0.16, *p* = 0.179; Figure 5B, bottom), but did in the StA condition (K-S D = 0.54, *p* = 0.001; Figure 5B, top). In this regard, the probability that a male would have a higher I-ACD score than a female in the control condition (PS_M-Con_ = 0.56 [0.48, 0.65]) was equivalent (Cliff’s delta = 0.13, *p* = 0.150) to that of a female having a higher I-ACD score than a male (PS_F-Con_ = 0.44 [0.35, 0.52]). However, after stereotype activation, there was a much higher probability that a randomly selected male would have a higher I-ACD score (i.e., consistently chose more difficult problems) than a randomly selected female (PS_M-StA_ = 0.81 [0.73, 0.86]; PS_F-StA_ = 0.19 [0.14, 0.27]; Cliff’s delta = 0.61, *p* < 0.001). As panel C of Figure 5 illustrates, these gender-based differences emerged from two statistically significant, similarly sized, but of opposite sign effects of stereotype activation in females and males. Thus, compared to the distributions of their respective control groups, the I-ACD scores’ distribution of the F-StA and M-StA were significantly shifted toward lower (K-S D = 0.26, *p* = 0.006; PS F-StA = 0. 37 [0.29, 0.45], PS F-Con = 0. 63 [0.55, 0.71], Cliff’s delta = −0.26, *p* = 0.004) and higher values (K-S D = 0.27, *p* = 0.003; PS M-Sta = 0.64 [0.56, 0.72], PS M-Con = 0.36 [0.28, 0.44], Cliff’s delta = 0.29, *p* = 0.001), respectively. Taken together, the results obtained indicate that the activation of stereotypes about a gender-based difference in math ability made males choose more difficult math problems, whereas it promoted the opposite effect in females.

##### 3.3.2.3. Does stereotype threat affect arithmetic accuracy?

Stereotypes’ activation did not seem to affect arithmetic accuracy. Thus, a two-way qANOVA (gender x experimental condition) comparing accuracy scores did not yield any statistically significant effect for any quartile (Table 5). No statistically significant differences were found when comparing the entire accuracy score distributions with Kolmogorov–Smirnov or Cliff’s delta tests (Table 6). Finally, no statistically significant differences were observed when accuracy scores were compared while controlling the possible effects of chosen difficulty by introducing the I-ACD scores as a covariate of no interest (Table 7).

**Table 5:** Quartile-based comparisons for arithmetic accuracy scores.

GROUP	Q25	Q50	Q75
M-Con	60.2	71.0	79.3
M-StA	61.4	72.1	80.4
F-Con	60.4	69.8	78.3
F-StA	60.1	70.9	78.0
	Gender, *p =* 0.881	Gender, *p* = 0.881	Gender, *p =* 0.417
	Condition, *p* = 0.856	Condition, *p* = 0.856	Condition, *p* = 0.856
	Interaction, *p* = 0.970	Interaction, *p* = 0.970	Interaction, *p* = 0.970

The table reports the outcome (*p*-value, quantile difference, and its corresponding 95% CI) of dyadic post-hoc comparisons conducted after having identified statistically significant gender x group interactions in quantile-based ANOVAs comparing arithmetic accuracy scores. Robust post-hoc comparisons were carried out with the *Qmcp* function and those reaching statistical significance after multiple comparisons correction with the Hochberg’s method are highlighted in bold. M-Con, males assigned to the control experimental condition; M-StA, males assigned to the stereotype activation condition; F-Con, females assigned to the control experimental condition; F-StA, females assigned to the stereotype activation condition.

**Table 6:** Whole distribution between-groups comparisons for arithmetic accuracy scores.

	K-S D	*p*-value	Cliff’s delta	*p*-value
M-StA vs. F-StA	0.11	0.653	0.07	0.470
M-Con vs. F-Con	0.08	0.931	0.04	0.643
M-Con vs. M-StA	0.10	0.686	0.04	0.620
F-Con vs. F-StA	0.06	0.983	0.02	0.831

The table summarizes the obtained statistics (D and delta), as well as the associated *p*-values for between-group comparisons on the whole distributions of accuracy scores conducted with the two-samples Kolmogorov-Smirnoff and the Cliff’s delta tests. All *value of p*s are corrected for multiple comparisons with the Hochberg’s method. K-S, two-samples Kolmogorov-Smirnoff test; M-Con, males assigned to the control experimental condition; M-StA, males assigned to the stereotype activation condition; F-Con, females assigned to the control experimental condition; F-StA, females assigned to the stereotype activation condition.

**Table 7:** Between-group median differences in accuracy scores after accounting for chosen difficulty variation.

Covariate value	Comparison	*p-*value	d^	95% CI
2	M-StA vs. M-Con	0.883	−1.63	[−11.90, −9.97]
	M-StA vs. F-Con	0.883	5.57	[−7.21, 15.27]
	M-StA vs. F-StA	0.883	5.20	[−6.89, 13.16]
	F-StA vs. M-Con	0.212	6.84	[−1.67, 12.78]
	F-StA vs. F-Con	0.883	0.37	[−8.49, 10.39]
	M-Con vs. F-Con	0.282	7.20	[−2.01, 15.94]
3	M-StA vs. M-Con	0.727	4.30	[−5.04, 15.69]
	M-StA vs. F-Con	0.727	2.18	[−6.20, 13.51]
	M-StA vs. F-StA	0.727	1.40	[−7.70, 16.51]
	F-StA vs. M-Con	0.727	−2.89	[−13.10, 11.47]
	F-StA vs. F-Con	0.727	−1.39	[−16.51, 7.70]
	M-Con vs. F-Con	0.727	−2.12	[−11.47, 9.31]
4	M-StA vs. M-Con	0.962	1.79	[−14.77, 11.43]
	M-StA vs. F-Con	0.962	−0.51	[−19.04, 13.91]
	M-StA vs. F-StA	0.962	4.91	[−14.39, 37.44]
	F-StA vs. M-Con	0.962	0.02	[−11.36, 35.71]
	F-StA vs. F-Con	0.962	−2.80	[−37.95, 12.01]
	M-Con vs. F-Con	0.962	−2.30	[−12.02, 37.95]
5	M-StA vs. M-Con	0.446	13.59	[−7.48, 30.19]
	M-StA vs. F-Con	0.446	14.18	[−7.58, 31.46]
	M-StA vs. F-StA	0.446	20.73	[−5.48, 33.72]
	F-StA vs. M-Con	0.738	−9.52	[−19.46, 0.40]
	F-StA vs. F-Con	0.738	−6.55	[−19.55, 6.46]
	M-Con vs. F-Con	0.738	0.59	[−7.28, 8.46]

The table displays the outcome (*p*-values, medians’ difference, and its corresponding 95% CI) of between-group comparisons for median accuracy scores conducted by means of a robust ANCOVA that included I-ACD scores as covariates of no interest. The column “covariate value” indicates the points at which the test statistic was evaluated. The reported *value of p*s are corrected for multiple comparisons with the Hochberg’s method. M-Con, males assigned to the control experimental condition; M-StA, males assigned to the stereotypes activation condition; F-Con, females assigned to the control experimental condition; F-StA, females assigned to the stereotypes activation condition; CI, confidence interval.

### 3.4. Relationship between math self-concept, the “influence” of pre-testing gender-related stereotypes, and M-MET performance

Robust moderation regression-based analyses revealed that the pre-testing science-gender stereotypes did only influence specific aspects of their M-MET performance in those individuals which pre-testing stereotypes were experimentally re-activated (StA condition). More specifically, it was observed that only the interaction between the imposed experimental condition and the IAT “influence” scores significantly predicted the overall MET scores (condition estimate = 1.17, *p* = 0.526, IAT estimate = 0.20, *p* = 0.939, interaction estimate = 14.50, *p* < 0.001). Given these results, the relationships between the M-MET components, IAT-“influence” scores and math self-concept scores were further and separately explored for each experimental condition with correlational methods.

**Figure 6:**
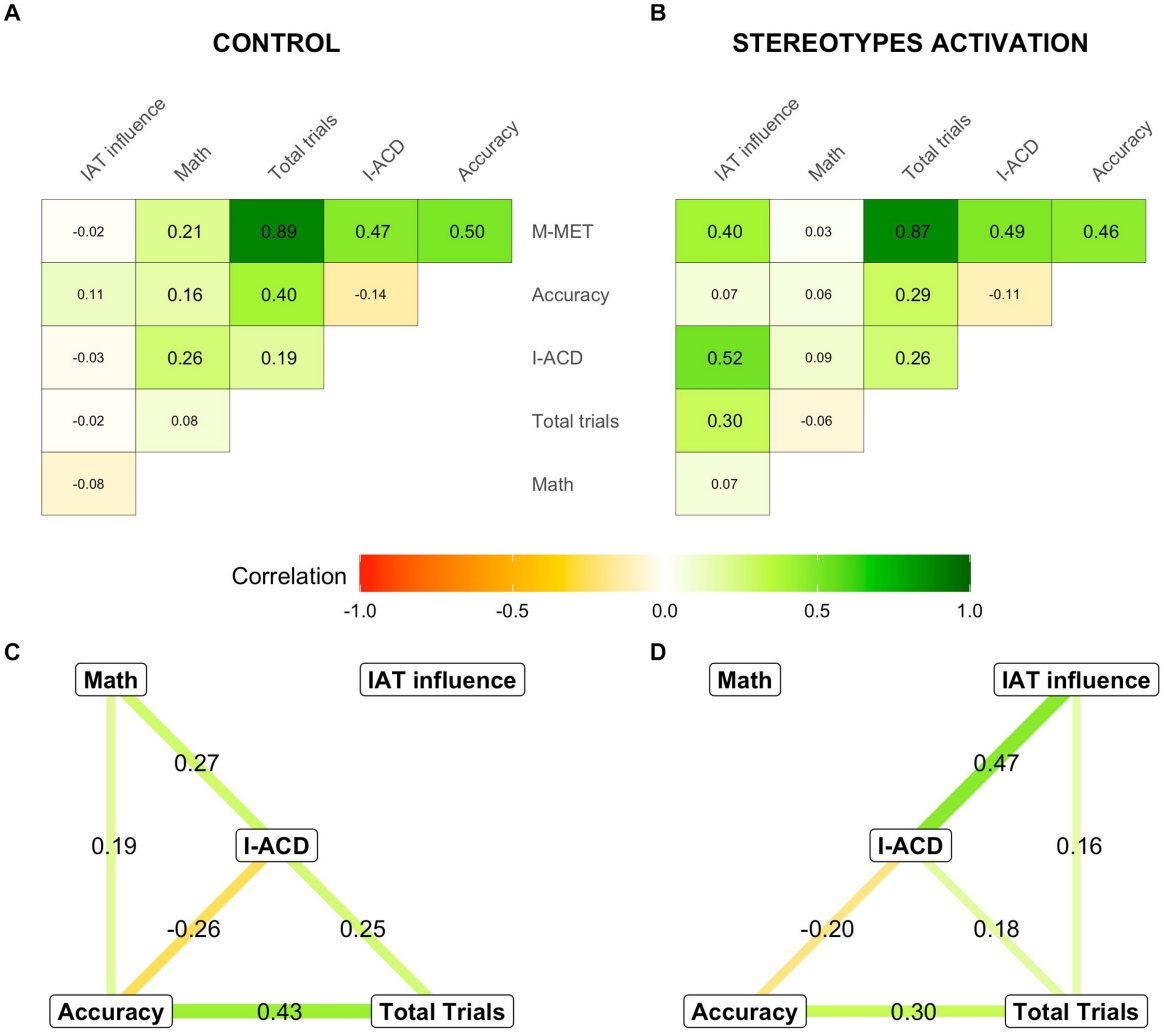
Relationship between self-competence in math, the “influence” of pre-existing gender-related stereotypes, and different index of performance in the M-MET. Panels **(A,B)** depict the zero-order Spearman’s correlation indexes between all the variables considered in this study (see below) observed in the participants (males and females) assigned to the control/stereotype reactivation condition, respectively. Correlation values are reported inside each cell (which are colored accordingly), and those reaching statistical significance (*p* < 0.05) are displayed with a larger font size. Panels **(C,D)** illustrate the LASSO-regularized partial correlation networks estimated for the control/stereotypes’ reactivation condition, respectively. In these panels, each node represents a variable and edges link pairs of variables that remain correlated after removing the influence of all the other variables in the model. The strength of these relationships is denoted by the edge thickness and color, but also by the reported edge weights. Math, Math self-concept scores; I-ACD, individual average chosen difficulty, M-MET, M-Met overall scores; IAT, implicit association test.

Panels A and B of Figure 6 depict the patterns of zero-order Spearman correlations between math self-concept, the IAT-“influence” scores, and the different measures obtained from the M-MET task in the control and the StA activation condition, respectively. As can be readily observed, the correlations between M-MET derived measures were very similar in both cases, whereas their correlations with the pre-testing math self-concept and IAT-“influence” scores clearly differed between experimental conditions. Thus, in the control condition (panel A), the overall M-MET, the I-ACD, and the accuracy scores were significantly correlated to math self-concept, but appeared to be unrelated to the IAT-“influence” scores. Conversely, in the StA condition (panel B), the number of completed trials as well as the overall M-MET and the I-ACD scores were significantly correlated with the IAT “influence,” but not with the math self-concept scores. Confirming and extending the results of our initial moderation analysis, the values of the correlations between the IAT “influence” scores and the M-MET scores (difference = 0.38 [0.21, 0.63], *p* < 0.001), I-ACD scores (difference = 0.49 [0.37, 0.74], *p* < 0.001), and the number of trials completed (difference = 0.28 [0.10, 0.54], *p* = 0.006) were significantly larger in the StA than in the control condition.

To assess the relationship between each possible pair of variables in each experimental condition while controlling the possible influence of all others and removing spurious correlations, LASSO-regularized partial correlation networks were estimated (Figures 6C,D). These networks exhibited a good fit to the data (control condition: CFI = 0.99, TLI = 0.97, RMSEA = 0.032; StA condition: CFI = 0.98, TLI = 0.96, RMSEA = 0.045) and confirmed that, although the relationships between the different M-MET components were very similar in both models, their regulation by pre-testing math self-concept and IAT-“influence” scores was very different in each experimental condition. More specifically, in the control condition (panel C), math self-concept was the only relevant predictor, and was directly associated with accuracy and I-ACD scores but, through these two mediators, also indirectly related to the number of completed trials. By contrast, in the StA condition (panel D), IAT-“influence” scores (but not math self-concept scores) were directly associated with the number of completed trials as well as with I-ACD scores, and, through both of them, also with accuracy scores. These networks proved to be significantly different (*M* = 0.443, *p* < 0.001) and statistically significant differences were observed at the edges, linking IAT “influence” to I-ACD scores (*p* < 0.001), the IAT “influence” scores to total trials (*p* = 0.022), and math self-concept to I-ACD scores (*p* = 0.055), but not in any of the edges linking the M-MET components between them (*p* > 0.2 in all cases). Taken together, these results suggest that, under ordinary circumstances, high/low math self-concept is associated with high/low self-selected difficulty (I-ACD scores) and arithmetic accuracy that result in high/low M-MET scores, respectively. In contrast, after a forced exposure to gender-related stereotypes, M-MET performance seems to get largely disengaged from the participants’ math self-concept, so their motivation to persist, self-selected difficulty, and performance in this task become dependent on the contents and strength of their pre-testing gender-science implicit associations.

**Figure 7:**
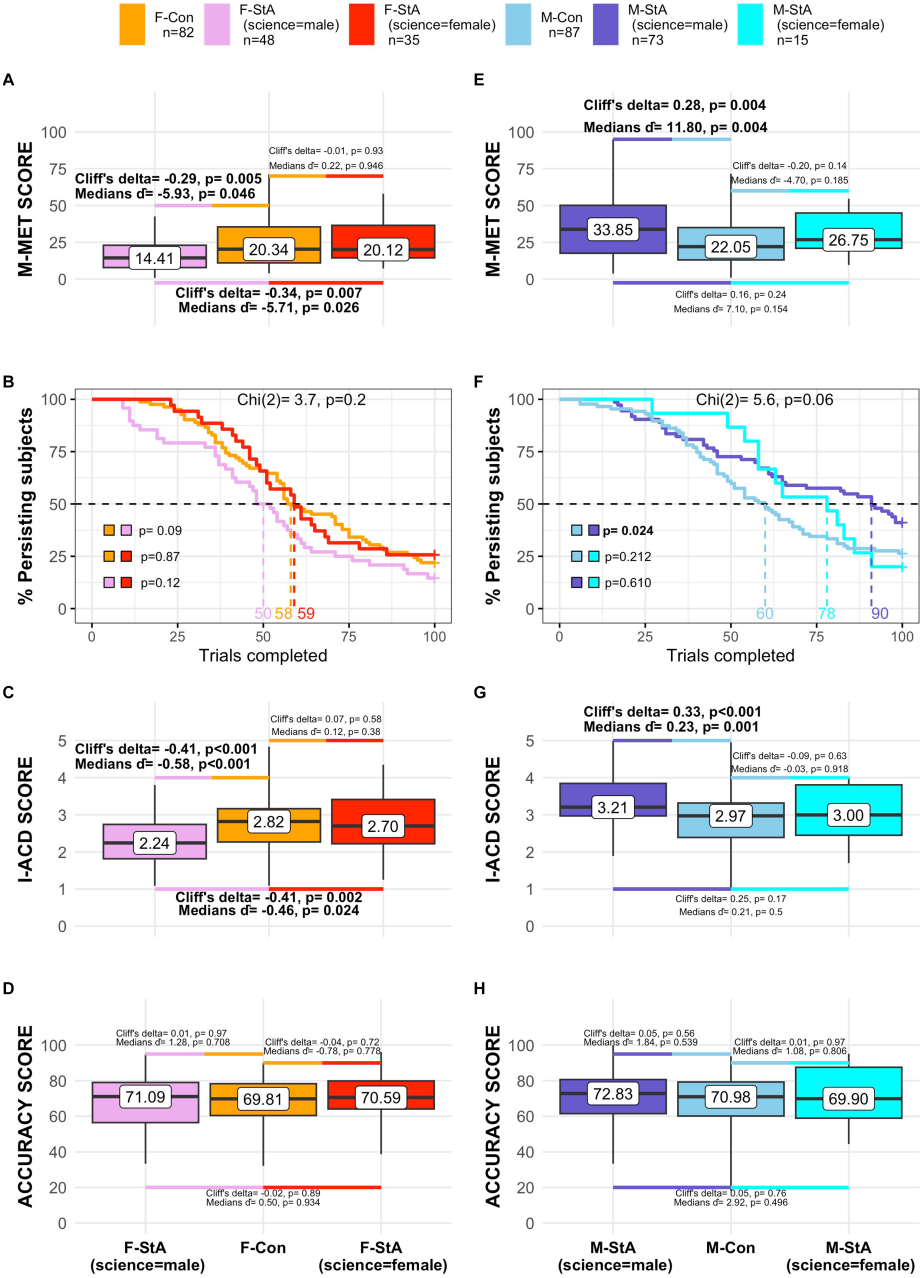
Effects of stereotypes’ reactivation in participants harboring stereotypical and counter-stereotypical “gender-science” implicit associations. The figure summarizes the M-MET performance after stereotypes’ reactivation of females (panels **A–D**) and males **(E–H)** holding stereotype-consistent associations (“science = male”) or stereotype-inconsistent (“science = female”) implicit associations (IAT D-score > 0 and IAT D-score < =0, respectively). Except for the total number of trials (panels **B,F**), comparisons between these subgroups of individuals and their respective control groups were conducted by calculating their median differences ( d^ ) and Cliff’s delta and the results are reported within the respective panels. Possible differences in task persistence (number of completed trials, panels **B,F**) were assessed by comparing the Kaplan -Meier survival curves with a Long-Rank test and subsequent dyadic comparisons (reported within the panels). Statistically significant effects (uncorrected *p* < 0.05) are highlighted in bold.

In this regard, additional intra-group analyses confirmed that only those individuals of M-StA and F-StA groups harboring stereotypical associations linking “science” to “male” -and, therefore, experiencing positive and negative “influences” from these associations, respectively- showed I-ACD and M-MET (but not accuracy) scores that were significantly higher/lower than their respective control groups, respectively (Figure 7). The within-gender differences in M-MET scores appeared to be more prominent in males than in females, probably because the number of completed trials was only significantly affected in males (Figures 7B,F). On the other hand, it is worth noting that these within-gender differences cannot be explained by any pre-existing difference in math self-concept between these male/female subgroups (Table 8).

**Table 8:** Comparison of the StA subgroups in math self-concept.

	*n*	Median	Value of *p*	d^	95% CI	Cliff’s delta
F-StA (“science = male”) Vs. F-Con	48	7	0.214	−0.67	[−1.5, 0.36]	Delta = −0.13, *p* = 0.22
	82	7.67				
F-StA (“science = female”) Vs. F-Con	35	7.56	0.862	0.11	[−0.80, 0.94]	Delta = 0.01, *p* = 0.95
	82	7.67				
F-StA (“science = male”) Vs. F-StA (“science = female”)	48	7	0.320	−0.56	[−1.55, 0.49]	Delta = 0.12, *p* = 0.36
	35	7.56				
M-StA (“science = male”) Vs. MCon	73	7.55	0.484	0.38	[−0.54, 0.92]	Delta = 0.03, *p* = 0.76
	87	7.17				
M-StA (“science = female”) Vs. M-Con	15	6.9	0.421	0.30	[−0.62, 1.65]	Delta = 0.16, *p* = 0.36
	87	7.17				
M-StA (“science = male”) Vs. M-StA (“science = female”)	73	7.55	0.220	0.68	[−0.43, 1.91]	Delta = 0.18, *p* = 0.29
	15	6.9				

The table displays the outcome (*p*-values, medians’ difference, and its corresponding 95% CI) of the median-based comparisons between control and StA subgroups regarding math self-concept. For each gender, the StA subgroups were constructed depending on whether individuals hold stereotypical “science-male / humanities-female” (IAT D scores >0) or counter-stereotypical “science-female / humanities-male” (IAT D-score < 0) associations. M-Con, males assigned to the control experimental condition; M-StA, males assigned to the stereotypes activation condition; F-Con, females assigned to the control experimental condition; F-StA, females assigned to the stereotypes activation condition; CI, confidence interval.

## 4. Discussion

The present study was designed to assess how experimentally activated stereotypes affect the performance and persistence of female and male engineering students in math tasks that require sustained effort (M-MET) and to which extent the effects of the induced threat are mediated by self-efficacy changes and moderated by the participants’ pre-testing stereotypes. Under the control condition (Con) both genders were found to exhibit similar M-MET performances. These between-gender similarities were apparent when attending to their overall M-MET scores (medians: 22.05 vs. 20.34) as well as to each of its subcomponents (medians completed trials: 60 vs.58, medians average difficulty: 2.97 vs. 2.82, and medians accuracy: 71 vs. 69.8%, respectively). The same level of similarity was observed for lower and higher levels of performance (Q25 and Q75) and also when considering the entire score distributions of each of these variables (Figures 2, 3, 5; Tables 3–7). Given that this is the first time that the M-MET has been used, these findings cannot be directly compared to those of any other study, although it is worth noting that they are similar to those reported for the original MET in which no gender-based differences were found (Engle-Friedman et al., 2003).

By contrast, between-gender differences emerged when participants were exposed to a stereotype threat (StA condition). In this case, females showed lower overall M-MET scores than males (Figure 2; Table 3) and these differences were increasingly larger across quartiles (Q75 > Q50 > Q25; Table 3). Moreover, the M-StA group exhibited significantly higher M-MET scores than the M-Con and F-Con groups at intermediate (Q50) and high (Q75) levels of performance, whereas the M-MET scores of the F-StA were significantly lower than those of both control groups at Q75 (Figure 2; Table 3). Taken together these results suggest that stereotype activation promoted opposite effects in the M-MET performance of male and female students, promoting gender-based differences that were not observed in the control condition. These effects can be suitably accounted for by the principles of the Stereotype Threat Theory (STT). More specifically, the reduced M-MET performance observed in the F-StA group can be understood as the result of a stereotype threat, whereas the increased performance in the M-StA group could potentially arise from a stereotype lift, a stereotype boost, or a combination of both kinds of effects. In this regard, it has been repeatedly observed that pre-exposure to explicit or implicit suggestions of women having lower abilities than men in math as well as cues indicating a gender-biased evaluation are able to promote a threat to female students that reduces their performance in math-related tasks (e.g., Spencer et al., 1999; Adams et al., 2006; Good et al., 2008; Picho and Schmader, 2018; for meta-analyses, see Nguyen and Ryan, 2008; Picho et al., 2013; Flore and Wicherts, 2015; Doyle and Voyer, 2016). On the other hand, although far less studied, it has also been shown that conditions triggering a threat for women can simultaneously promote a stereotype lift effect in men, indirectly enhancing their math performance (e.g., Spencer et al., 1999; Eriksson and Lindholm, 2007; Good et al., 2008; Picho and Schmader, 2018; for a meta-analysis, see Walton and Cohen, 2003). The enhanced performance observed in the M-StA group could also be due to a stereotype boost directly elicited by their exposure to male-favoring stereotyped information, but this seems less likely because, opposite to the stereotype threat and lift phenomena, stereotype boost is less likely to occur when stereotypes are explicitly activated (Shih et al., 2012).

Even though numerous studies have illustrated the effects of stereotype threat (and related phenomena) on cognitive performance, not all studies have found these stereotypes’ effects (Stoet and Geary, 2012; Flore and Wicherts, 2015; Flore et al., 2018) and far less is known about *how* they take place and *who* may be more prone to experience them (Pennington et al., 2016). Therefore, the present study also attempted to provide more specific information on these two questions.

Regarding the “how” question, the three separate subcomponents of M-MET scores (number of completed trials, chosen difficulty, and arithmetic accuracy) were separately analyzed, hence obtaining information about the consequences of the experimentally imposed stereotype threat in each of the three main processes underlying M-MET performance (that were interpreted as indicators of persistence/motivation, self-confidence while performing the task, and arithmetic accuracy, respectively). Given that the M-StA group completed more trials (Figure 3) and chose more difficult problems than all other groups (I-ACD scores; Figures 4, 5; Table 4), it can be tentatively concluded that their enhanced M-MET performance stemmed from two complementary effects in motivation/persistence and in-task self-confidence. On the other hand, the F-StA group showed lower I-ACD scores than all the other groups without significantly differing in any other M-MET component, suggesting that the decreased M-MET performance of the F-StA group was primarily due to a reduction of in-task self-confidence during task performance (Figures 4, 5; Table 4). Taken together, these results may be interpreted as indicating that the effects of stereotype threat on M-MET performance were primarily -but not exclusively- conveyed through in-task self-confidence changes. In this regard, it is worth noting that STT assumes self-confidence as a mediator of stereotype effects (e.g., Steele, 1997; Walton and Cohen, 2003; Schmader et al., 2008). Moreover, several studies have reported that stereotype threat reduces task-specific measures of self-confidence in women facing math tasks (Good et al., 2008; Franceschini et al., 2014) while increasing their negative thoughts (Cadinu et al., 2005; Beilock et al., 2007; Schmader et al., 2008), and their anxiety and feelings of dejection (Spencer et al., 1999; Keller and Dauenheimer, 2003; Osborne, 2007; Johns et al., 2008). Stronger and bidirectional, albeit indirect, evidence of a mediatory role of in-task self-confidence also comes from studies assessing the effects of gender-related stereotypes on male and female performance in another highly gender-stereotyped cognitive domain, mental rotation (e.g., Estes and Felker, 2012; Sanchis-Segura et al., 2018).

To further explore the possible mediatory role of in-task self-confidence in M-MET performance and also obtain information about “who” is more affected by the stereotypes’ activation, *across* gender effects were assessed with correlational methods and mediation analyses. To do so, IAT D-scores were first transformed into IAT “influence” scores (see Section 2.3 for details). These scores quantify the strength of the association between “science” and each participant’s own gender category and, therefore, the expected positive/negative impact of these implicit associations on the participants’ selves (Nosek and Smyth, 2011; Steffens and Jelenec, 2011) and not just the stereotypical/counter-stereotypical direction of their contents.

As could be expected, in the absence of a stereotype activation, neither M-MET scores nor any of its components appeared to be “influenced” by the participants’ gender-science implicit associations. Instead, overall M-MET, accuracy, and, even more so, I-ACD scores were directly correlated to the participants’ pre-test math self-concept (Figure 6A). Partial correlation networks (Figure 6C) confirmed these exploratory observations and revealed that the relationship between math self-concept and MET scores was directly mediated through I-ACD and, to a lower extent, by accuracy scores. Moreover, through these two mediators, math self-concept also indirectly increased task persistence. That is, participants that perceived themselves as more competent in math before the test were also the ones that felt more self-confident while performing this task and, through several converging processes, ended up obtaining higher M-MET scores. These findings align with the predictions of Bandura’s social cognitive theory (Bandura, 1977; Bandura, 1986), the Eccles’ expectancy-value model (Eccles, 1983; Wigfield and Eccles, 2000), and SCCT (Lent et al., 1994) and, therefore, with the results of previous studies showing that math self-concept is positively associated to math performance, academic achievement, and persistence in math-related activities (e.g., Multon et al., 1991; Malmivuori, 2006; Brown et al., 2008; Marsh et al., 2009; Parsons et al., 2009; Lee et al., 2015). Moreover, these results also confirm that, as has been previously proposed (Pajares and Miller, 1994; Pajares, 1996), task-specific indexes of self-efficacy have higher and a more direct predictive value on math performance and persistence than math self-concept and other general measures of math self-efficacy.

By contrast, M-MET scores in the StA condition became largely independent on math self-concept and clearly associated to the participants’ implicit gender-science associations (Figure 6B). Partial correlation networks (Figure 6D) revealed that the effects of the participants’ implicit gender-science associations on M-MET scores were primarily conveyed through I-ACD scores. Thus, the higher the participants implicitly associated science with their own gender (that is, the higher the positive “influence” of the participants’ implicit associations), the higher their self-confidence during task performance, and the higher their final M-MET scores. Positive “influences” of the participants’ implicit associations were also directly correlated to the number of completed trials (Figure 6D) and, although this effect appeared to be smaller, it was reinforced by converging indirect effects mediated by I-ACD and accuracy scores, all of which contributed to enhance the overall M-MET scores. These findings are in agreement with the principles of cognitive consistency (Greenwald et al., 2003; Cvencek et al., 2011) and, therefore, with empirical evidence showing that implicit gender-related associations can affect math self-efficacy, achievement, and engagement/persistence (e.g., Nosek et al., 2002a,b; Nosek and Smyth, 2011; Steffens and Jelenec, 2011; Franceschini et al., 2014). Moreover, our results seem to be also in line with studies suggesting that stereotype threat undermines the achievement, interest, and persistence of female STEM students in in math/STEM-related activities through a reduction of self-confidence and self-efficacy beliefs (Brown et al., 2008; Wright et al., 2013; Deemer et al., 2014; Lee et al., 2015; Lent et al., 2015; Lin and Deemer, 2021) but probably also through other behavioral/cognitive processes (Woodcock et al., 2012; Thoman et al., 2013; Lewis and Sekaquaptewa, 2016).

To better characterize the modulatory role of gender-science implicit associations on the stereotype threat effects, within-gender comparisons between participants exposed to the StA condition but experiencing distinct (positive vs. negative) “influences” from their implicit associations in M-MET scores were conducted (Figures 7A,E). The results of these comparisons suggest that stereotype threat solely promoted a statistically significant decrease of the M-MET scores in those female students associating “science” with “male” (median IAT D-score = 0.34) and, therefore, suffering a negative “influence” from their implicit associations (median IAT “influence” = −0.34). Complementarily, stereotype activation only promoted a statistically significant increase of the M-MET scores in those male students implicitly associating “science” with “male” (median IAT D-score = 0.65) that is, in those males receiving positive “influences” from their implicit associations (median IAT “influence” = 0.65). By contrast, the M-MET scores of males and females that did not associate “science” with “male” did not significantly differ from those of their respective control groups. More specifically, this subgroup of females exhibited a counter-stereotypical “female-science” association (median IAT D-score = −0.37) that promoted a positive (median IAT “influence” = 0.37) and “protective” influence against the detrimental effects of the stereotype threat without triggering any stereotype reactance effect (Kray et al., 2001), hence showing M-MET scores virtually indistinguishable from those of the F-Con group. By contrast, the male subgroup that did not seem to associate “science” to any gender (median IAT D-score = −0.08) did not seem to receive any “influence” from their implicit associations (median IAT influence = −0.08) but seemed to “profit” from the male-encouraging stereotype activation, exhibiting M-MET scores that were slightly higher than those of the M-Con group (for similar instructions’ effects, see Moè and Pazzaglia, 2006 and Sanchis-Segura et al., 2018).

In agreement with the results of our correlational analyses (Figure 6), the same pattern of within-gender effects was observed on I-ACD scores (Figures 7C,G) and, to a reduced extent, in the number of completed trials (Figures 7B,F), but not in accuracy scores (Figures 7D,H). Therefore, the results of the between, across, and within gender analysis seem to confirm that the effect of stereotype reactivation on M-MET scores arise from changes in self-confidence and task persistence, but also that these effects solely occur or, at least, are more prominent in individuals holding stereotypical implicit gender-science associations. In this way, the results of the present study seem to confirm and extend those of previous reports showing that the effects of stereotype threat and stereotype lift are more prominent in individuals harboring stereotype-consistent implicit associations (Franceschini et al., 2014; Galdi et al., 2014), are probably mediated by in-task self-confidence changes (Franceschini et al., 2014), and may finally affect math-related achievements and the intentions of persisting or quitting math-related activities and math-related studies (Steele and Aronson, 1995; Von Hippel et al., 2011; Woodcock et al., 2012; Thoman et al., 2013).

## 5. Conclusions and implications

The present study provides experimental evidence showing that (at least, some) female engineering students are threatened by stereotypes about women’s math/science abilities. This threat seems to undermine their self-confidence, making them opt for less challenging options, and finally decreasing their achievement in high-demanding math activities. Thus, our findings confirm and extend slowly accumulating evidence showing that stereotype threats may act as a contextual barrier to women’s STEM career development (Deemer et al., 2014; Cadaret et al., 2017) and that gender stereotypes do not only make it less likely for women to initially choose engineering and other STEM studies, but can also create adverse environmental conditions for women already enrolled in these studies (Blackburn, 2017; Clark et al., 2021). These hostile conditions may reduce the self-efficacy, achievements, and engagement of female students (Eddy and Brownell, 2016; Blackburn, 2017), making their academic experiences less rewarding and more distressing (Eddy and Brownell, 2016; González-Pérez et al., 2022), and finally increasing their chances of abandoning the field or switching to less math-intensive (i.e., non-STEM) educational/professional options (Isphording and Qendrai, 2019; Clark et al., 2021; González-Pérez et al., 2022).

However, our study also shows that gender stereotypes do not only affect women’s performance. In fact, similar or even stronger effects but of the opposite sign (i.e., increased self-confidence and motivation) were observed in (at least, some) male engineering students. Traditionally, these effects have received less attention (Walton and Cohen, 2003; Shih et al., 2012) but they are also relevant as they show that stereotypes affect men and women differently but through similar processes (Cheryan and Plaut, 2010) and that self-affirming conditions can maximize students’ potential and performance (Walton and Cohen, 2003; Gawronski et al., 2008), hence providing the basis for designing effective interventions that can equally benefit female and male students (Good et al., 2008; Forbes and Schmader, 2010; Miyake et al., 2010; Walton et al., 2015). Moreover, these effects seem to indicate that interventions should not be solely focused on counteracting the stereotypes held by the disfavored groups (in this case, female STEM students) but should also probably try to change the environmental cues, cultural values, and other people’s beliefs that may indirectly promote them (Cheryan and Plaut, 2010; Walton et al., 2015; Rincón and George-Jackson, 2016; Blackburn, 2017).

Finally, our study shows that stereotype effects were not uniform, neither among female nor among male students, but very much moderated by their implicit and pre-existing “gender-science” associations. This observation is important, not only because it contributes to understanding the preconditions under which stereotypes’ effects emerge (Pennington et al., 2016; Picho-Kiroga et al., 2021), but also because it highlights that gender categories are far from being homogenous and, therefore, that characterizing these heterogeneous groups through averages, or any other single estimate can be very misleading. In this regard, we advocate to replace analytical strategies based on comparisons between men vs. women averages (which far too often are used to make unwarranted generic statements and conclusions about *all* women and *all* men) by more complex and nuanced ones able to also offer information about within and across gender effects. On the other hand, we also propose that the evaluation of pre-existing implicit gender-related associations may serve to identify individuals at higher risk of suffering the detrimental effects of stereotype threat on performance and career decisions in highly stereotyped academic domains such as math and other “hard” sciences.

## 6. Limitations

The present study is not devoid of limitations. In this regard, the main limitations that we see in our study are:

1. Experimental studies -as the present one- have the strength of allowing us to isolate and manipulate specific factors in order to clarify their role in multicausal and complex phenomena. However, experimental studies are conducted under conditions that are, to some extent, artificial and cannot fully model the complexity of the studied phenomena. Therefore, the ecological validity of experimental studies is necessarily limited and the information provided by this kind of studies must be regarded as complementary and not substitutive of the correlational evidence obtained in real world situations (Aronson and Dee, 2012).
2. The present study was conducted employing a large, gender-balanced, and very homogenous sample (engineering students). While this methodological decision was adequate and convenient in many ways, it could further reduce the generalization of the obtained findings and conclusions. Thus, as hinted in the introduction section, the math self-efficacy and gender-science implicit associations of engineering students probably do not correctly represent those of other (i.e., non-STEM) university students or of the general population. On the other hand, although our sample was large and we used robust statistics that enhanced statistical power, its size could have still been sub-optimal for some analyses conducted with some specific subgroups of participants.
3. To our knowledge, this is the first study using a modified version of the MET task developed by Engle-Friedman et al. (2003). This task allowed us to different measures (overall scores, total number of trials completed, percent of correctly solved problems, and level of difficulty chosen in each trial) that were interpreted as indexes of performance, persistence, arithmetic accuracy, and in-task self-confidence, respectively. However, although we think that our interpretation of these indexes is reasonable, there might be other alternative ones (especially in the case of self-selected difficulty, which could also be measuring more than one single construct). Therefore, future studies should be aimed to better characterize these different aspects of the M-MET task used in the present study and to validate their provisional interpretation.

## Data availability statement

The datasets presented in this article are not readily available because any data sharing should be approved by the Ethics Standards Committees of the Universitat Jaume I. Requests to access the datasets should be directed to CS-S (csanchis@uji.es).

## Ethics statement

The studies involving human participants were reviewed and approved by the study was approved by the Ethics Standards Committees of the Universitat Jaume I. The patients/participants provided their written informed consent to participate in this study.

## Author contributions

CF and CS-S designed and conceptualized the study. AS-T and SF-E preprocessed the data with which CS-S conducted the statistical analyses. CS-S and CF wrote the manuscript. All authors participated in the experimental sessions, contributed to the manuscript revision and read, edited, and approved the submitted version.

## Funding

This research was supported by a grant (PID2019-106793RB-I00/AEI/10.13039/501100011033) provided by Ministerio de Ciencia e Innovación to CF and CS-S and a grant (UJI B2020-02) awarded to CF and CS-S. SF-E was supported by an FPI grant from UJI (PREDOC/2020/22). These funding sources did not play any role in designing the study or in the collection, analysis, and interpretation of the data.

## Conflict of interest

The authors declare that the research was conducted in the absence of any commercial or financial relationships that could be construed as a potential conflict of interest.

## Publisher’s note

All claims expressed in this article are solely those of the authors and do not necessarily represent those of their affiliated organizations, or those of the publisher, the editors and the reviewers. Any product that may be evaluated in this article, or claim that may be made by its manufacturer, is not guaranteed or endorsed by the publisher.

## Footnotes

^1^https://www.millisecond.com/download/library/iat/genderscienceiat

^2^https://www.millisecond.com/download/library/effortexpenditureforrewardtask

^3^https://www.millisecond.com/download/library/mathefforttask
